# Increasing lysine level improved methanol assimilation toward butyric acid production in *Butyribacterium methylotrophicum*

**DOI:** 10.1186/s13068-023-02263-w

**Published:** 2023-01-17

**Authors:** Jing Wang, Yang Liao, Jialun Qin, Chen Ma, Yuqi Jin, Xin Wang, Kequan Chen, Pingkai Ouyang

**Affiliations:** State Key Laboratory of Materials-Oriented Chemical Engineering, College of Biotechnology and Pharmaceutical Engineering, Nanjing Tech University, Nanjing, 211816 Jiangsu China

**Keywords:** Methylotrophy, *B. methylotrophicum*, Lysine, Butyric acid

## Abstract

**Background:**

Methanol, a promising non-food fermentation substrate, has gained increasing interest as an alternative feedstock to sugars for the bio-based production of value-added chemicals. *Butyribacterium methylotrophicum,* one of methylotrophic-acetogenic bacterium, is a promising host to assimilate methanol coupled with CO_2_ fixation for the production of organic acids, such as butyric acid. Although the methanol utilization pathway has been identified in *B. methylotrophicum*, little knowledge was currently known about its regulatory targets, limiting the rational engineering to improve methanol utilization.

**Results:**

In this study, we found that methanol assimilation of *B. methylotrophicum* could be significantly improved when using corn steep liquor (CSL) as the co-substrate. The further investigation revealed that high level of lysine was responsible for enhanced methanol utilization. Through the transcriptome analysis, we proposed a potential mechanism by which lysine confers improved methylotrophy via modulating NikABCDE and FhuBCD transporters, both of which are involved in the uptake of cofactors essential for enzymes of methanol assimilation. The improved methylotrophy was also confirmed by overexpressing *NikABCDE* or *FhuBCD* operon*.* Finally, the de novo synthetic pathway of lysine was further engineered and the methanol utilization and butyric acid production of *B. methylotrophicum* were improved by 63.2% and 79.7%, respectively. After an optimization of cultivation medium, 3.69 g/L of butyric acid was finally achieved from methanol with a yield of 76.3%, the highest level reported to date.

**Conclusion:**

This study revealed a novel mechanism to regulate methanol assimilation by lysine in *B. methylotrophicum* and engineered it to improve methanol bioconversion to butyric acid, culminating in the synthesis of the highest butyric acid titer reported so far in *B. methylotrophicum*. What’s more, our work represents a further advancement in the engineering of methylotrophic-acetogenic bacterium to improve C1-compound utilization.

**Graphical Abstract:**

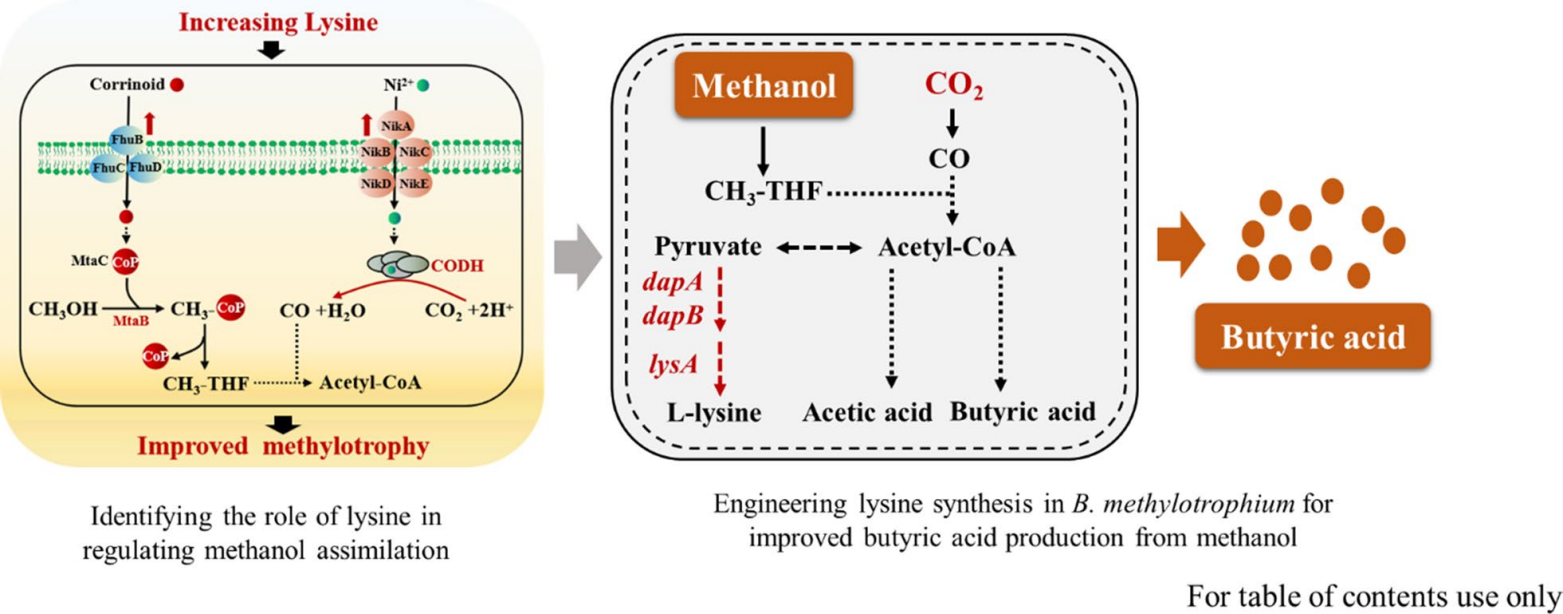

**Supplementary Information:**

The online version contains supplementary material available at 10.1186/s13068-023-02263-w.

## Background

The microbial assimilation of one-carbon (C1) compounds has been argued for as a promising technological approach to meet the growing challenges of the global climate change and energy resource scarcity [1]. As one of C1 compounds, methanol has attracted particular attention due to its abundance and liquid state. More importantly, methanol represents the highest electron content in all liquid C1 compounds and can also be easily produced from the greenhouse gases methane and CO_2_ [2–4], which makes it more promising as a non-food fermentation substrate to replace conventional sugars [5–7]. In recent years, efforts on the bioconversion of methanol into fuels and value-added chemicals have therefore intensified by the engineering of the native methylotrophs for improved methylotrophic phenotype, or development of entirely new methylotrophic organisms [8–12].

Native methylotrophs are the ideal host for methanol-based biomanufacturing as their ability to grow on methanol as the sole carbon and energy source. Methylotrophic-acetogenic bacterium, such as *Acetobacterium woodii* (*A. woodii*), *B. methylotrophicum*, *Eubacterium limosum* (*E. limosum*), *Sporomusa ovata*, *or Moorella thermoacetica*, are a group of obligate anaerobes that could utilize various C1 compounds (CO_2_, CO, methanol and formate) as growth substrates [13–15]. *B. methylotrophicum* was one of represented methylotrophic-acetogenic bacterium with the ability to convert methanol into butyric acid, a four-carbon carboxylic acid that could be widely used in the food, pharmaceuticals, and polymer industries. The methanol utilization pathway has been identified in *B. methylotrophicum*, where methanol assimilation was coupled with CO_2_ fixation through the methyltransferase systems and the carbonyl branch of the Wood–Ljungdahl pathway (WLP) [16]. The characteristic of co-utilization of methanol and CO_2_ to produce butyric acid by *B. methylotrophicum* has made it more potential to serve as a chassis for methanol bioconversion. Therefore, the further improvement in the methanol conversion efficiency of *B. methylotrophicum* is highly desired.

Recent efforts have been made to develop the genetic tools for engineering methylotrophic-acetogenic bacterium [17]. In the previous work, the plasmids with the stable replication origins, the functional promoters as well as the efficient electroporation protocols have been also developed for the engineering of *B. methylotrophicum* [16]. Based on these genetic tools, the genes encoding the methyltransferase systems (*mtaA*, *mtaB* and *mtaC*) in *B. methylotrophicum* were overexpressed, and the biomass yield, methanol consumption, and butyric acid production were increased by 1.25-fold, 1.69-fold, and 1.38-fold, respectively [16]. What’s more, several valuable chemicals, such as acetone and lactate have been produced through the metabolic engineering of methylotrophic-acetogenic bacterium [18, 19]. However, little knowledge was currently known on the understanding of regulation targets to improve methanol utilization of the methylotrophic-acetogenic bacterium. Adaptive laboratory evolution was thus employed for enhancing methylotrophy [20]. The understanding of metabolic regulation targets of methylotrophic-acetogenic bacterium is urgently required in improving acetogenic conversion of methanol.

As an important nutrient component, we hypothesized that the nitrogen source might affect methanol assimilation of methylotrophic-acetogenic bacterium as observed in other native methylotrophs [21, 22]. With *B. methylotrophicum* as a representative, the effect of different nitrogen source on methanol utilization was evaluated, and corn steep liquor (CSL) was found to be more superior in enhancing methanol utilization. Subsequently, high levels of lysine in CSL medium were identified to be responsible for the improved methylotrophy of *B. methylotrophicum*. Transcriptome analysis revealed that the addition of lysine up-regulated eight ABC transporters (*NikABCDE* and *FhuBCD*), involving in the transport of the essential cofactors for the enzymes in the methanol assimilation pathway. The improved methylotrophy of *B. methylotrophicum* was further confirmed by the overexpression of *NikABCDE* or *FhuBCD* operon, suggesting a potential molecular mechanism by which lysine improves methanol utilization of *B. methylotrophicum*. Finally, the de novo synthetic pathway of lysine was engineered to enhance the methanol bioconversion, and the highest reported titer of butyric acid from methanol was achieved*.*

## Results

### Corn steep liquor is a superior nitrogen source compared to yeast extract in enhancing methanol assimilation

In addition to carbon source, nitrogen source is another key nutrient that affects cell growth and product formation. In native methylotrophic *P. pastoris*, nitrogen source was reported capable of regulating the gene expression in methanol utilization pathway [21]. In synthetic methylotrophic *E. coli*, the addition of yeast extract could also enhance methanol assimilation [23]. Thus, we hypothesized that the types of nitrogen sources contributed differently to the methanol assimilation of *B. methylotrophicum*. To test it, different kinds of nitrogen sources including peptone, corn steep liquor, beef extract, ammonium chloride and ammonium sulphate were supplemented in the modified DSM 135 medium with 200 mM methanol to replace the yeast extract with the equal nitrogen content.

**Fig. 1:**
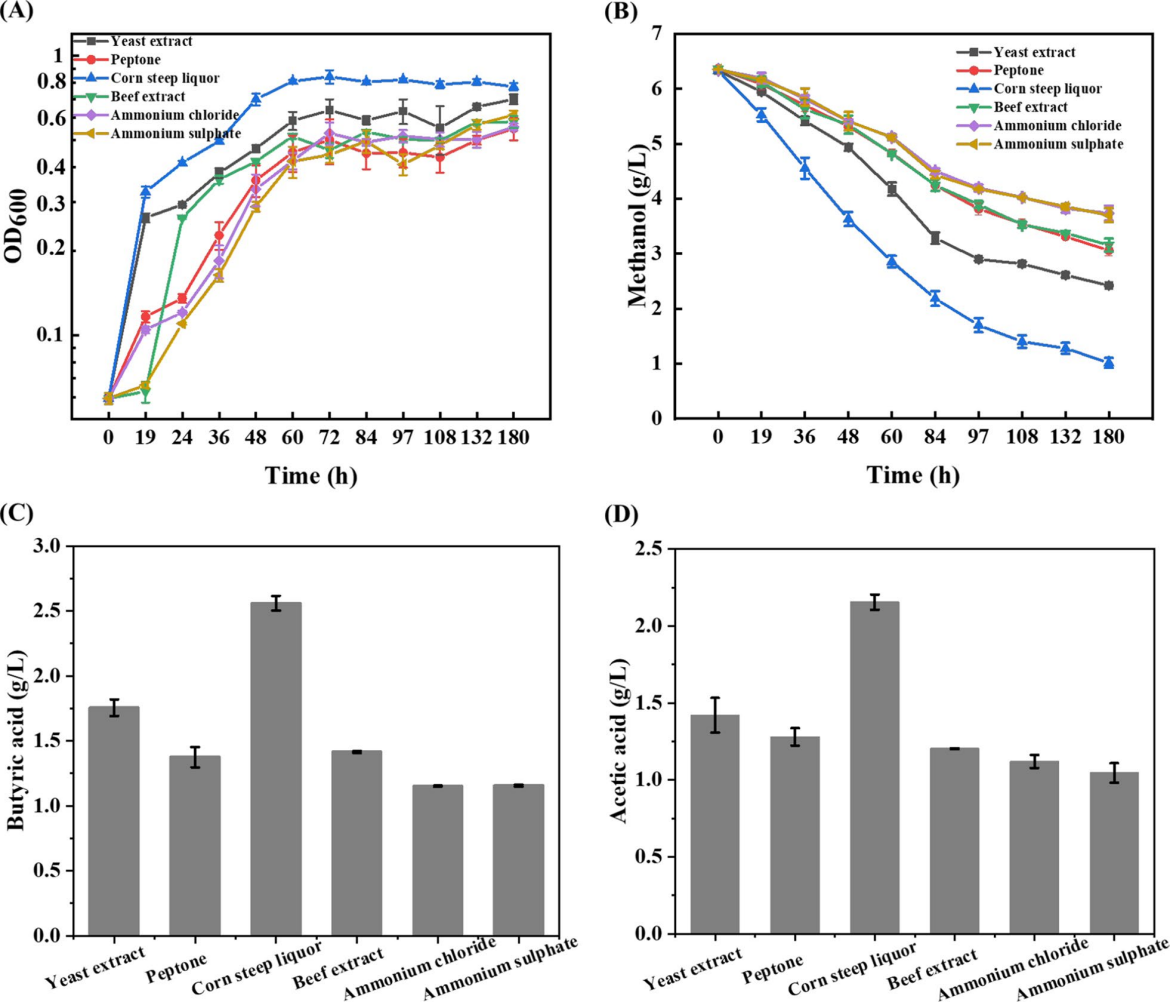
Evaluating the effect of nitrogen source on the methanol fermentation phenotype of *B. methylotrophicum.* **A** The growth of *B. methylotrophicum* in medium supplemented with different nitrogen sources. **B** The methanol consumption of *B. methylotrophicum* in medium supplemented with different nitrogen sources. **C** The butyric acid production of *B. methylotrophicum* in medium supplemented with different nitrogen sources. **D** The acetic acid production of *B. methylotrophicum* in medium supplemented with different nitrogen sources

As shown in Fig. 1, when the yeast extract was replaced by peptone, beef extract or inorganic nitrogen source of ammonium chloride and ammonium sulphate, the cell growth and methanol consumption were significantly decreased. With ammonium sulphate as an example, the final biomass and methanol consumption rate were decreased by 43% and 32%, respectively (Fig. 1). On the contrary, the use of corn steep liquor increases the methanol utilization rate of *B. methylotrophicum* by 35.9% (Fig. 1b). The final biomass yield in corn steep liquor medium was increased by 56.5% with a yield of 0.248 g_DCW_/g_MeOH_ (Fig. 1a). The butyric acid formation in different nitrogen source was consistent with the methanol consumption. In corn steep liquor medium, 2.56 g/L butyric acid was produced with a yield of 56.0%, a 1.07-fold higher than that in yeast extract medium, while 2.16 g/L acetic acid was accumulated (Fig. 1c, d). The results indicated that corn steep liquor was a superior nitrogen source for *B. methylotrophicum* to grow in methanol.

### Lysine was identified to be responsible for enhanced methylotrophy in *B. methylotrophicum*

Free amino acid is the main content of organic nitrogen source. To elucidate the potential molecular mechanism by which the corn steep liquor (CSL) was benefit for methanol assimilation of *B. methylotrophicum* compared to yeast extract (YE), the amino acid composition in CSL medium and YE medium was comparatively investigated during the fermentation process. The culture broth was sampled at 0 h, 36 h (middle exponential phase), 60 h (later exponential phase), and 84 h (stationary phase), respectively. Through the GC/MS analysis, 15 kinds of amino acids were detected including Gly, Ala, Lys, Asn, Thr, Glu, Asp, Val, Ile, Ser, Tyr, Pro, and Phe. The levels of the 15 amino acids were all gradually decreased with the fermentation both in the CSL medium and YE medium (Additional file 1: Fig. S1).

**Fig. 2:**
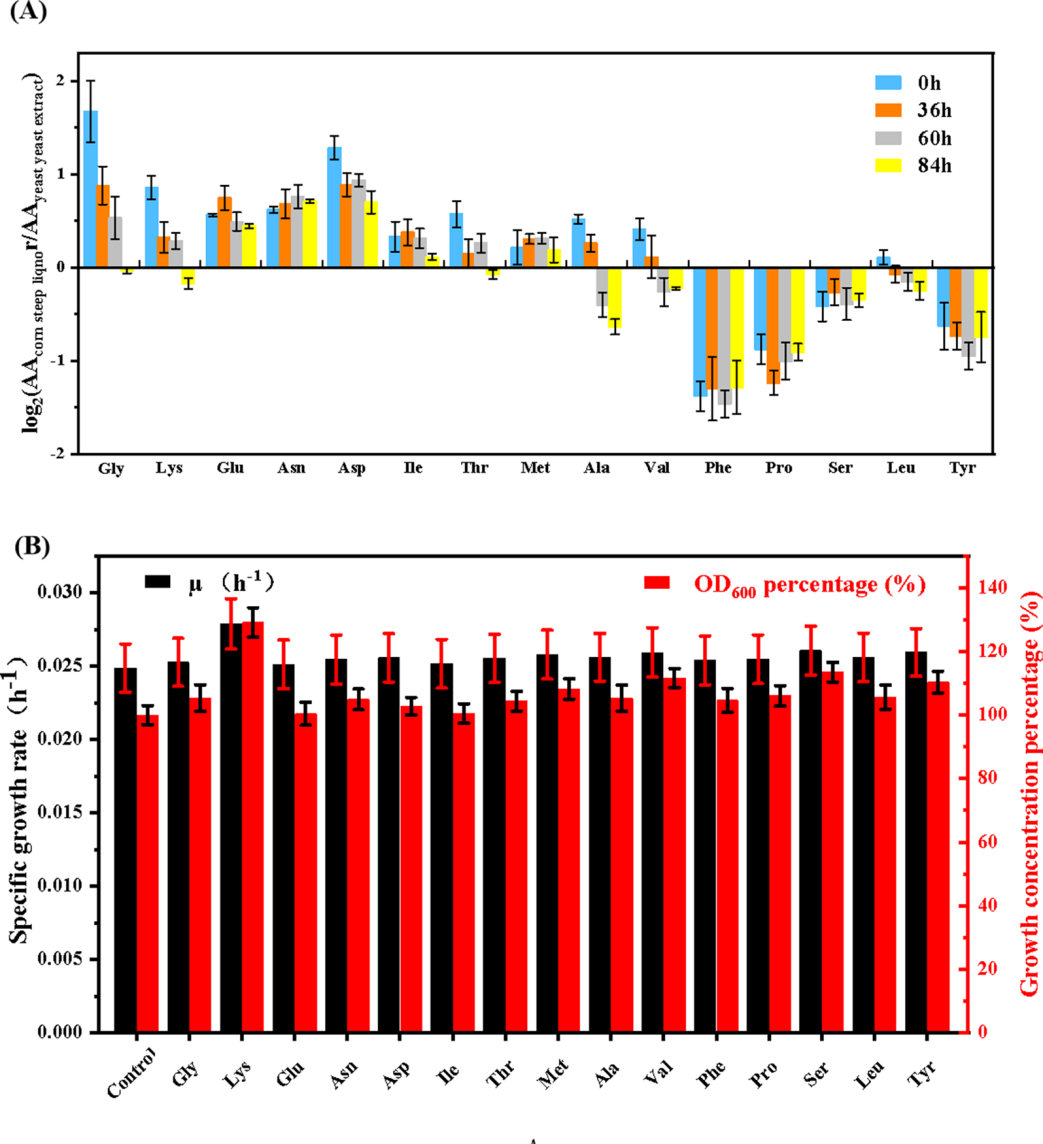
The identification of the key factors by which corn steep liquor is superior to yeast extract in improving methylotrophy of *B. methylotrophicum.* **A** The difference of amino acid component between corn steep liquor medium and yeast extract medium in different fermentation stage. All ratio values were log-transformed (base 2) for the fold change of the relative abundance of each amino acid in CSL medium relative to that in YE medium in different fermentation stage. **B** 15 kinds of amino acids (5 mM) were supplemented to the medium for their effects on methylotrophy of *B. methylotrophicum*, respectively

Among them, the content of 11 amino acids in CLS medium was initially higher than those in YE medium, which are Gly, Lys, Glu, Asn, Asp, Ile, Thr, and Met respectively (Fig. 2a). These results indicated the advantage of CLS in supplying free amino acids. During the whole fermentation process, the contents of Glu, Asn, Asp, Ile, Thr and Met in CLS medium were always higher than those in YE medium, while the contents of Phe, Pro, Ser and Leu in CLS medium were always lower than those in YE medium (Fig. 2a). For the amino acids with the higher level in CLS medium, Gly, Lys, and Asn represented the most significant difference. In addition, the difference of Gly, Lys, Ala and Val in CLS medium and YE medium changed most significantly during the whole fermentation process (Fig. 2a). We speculated that these significantly different amino acids with higher content in CSL medium might be related to enhanced methanol assimilation in *B. methylotrophicum.*

To further identify the certain amino acids important for enhanced methanol metabolism in *B. methylotrophicum*, Gly, Lys, Ala, Val, and Asn were added into the medium to evaluate their effects, while the other 9 amino acids were also comparatively analyzed. From the results shown in Fig. 2b, when methanol was used as a sole carbon source, the specific growth rate and the final biomass of *B. methylotrophicum* were improved by 12.1% and 29.2% with the addition of Lys, while the addition of Gly, Val and Ala showed a moderately increase in final biomass. Based on these results, lysine was determined as an important factor for regulating methanol assimilation of *B. methylotrophicum.*

**Fig. 3:**
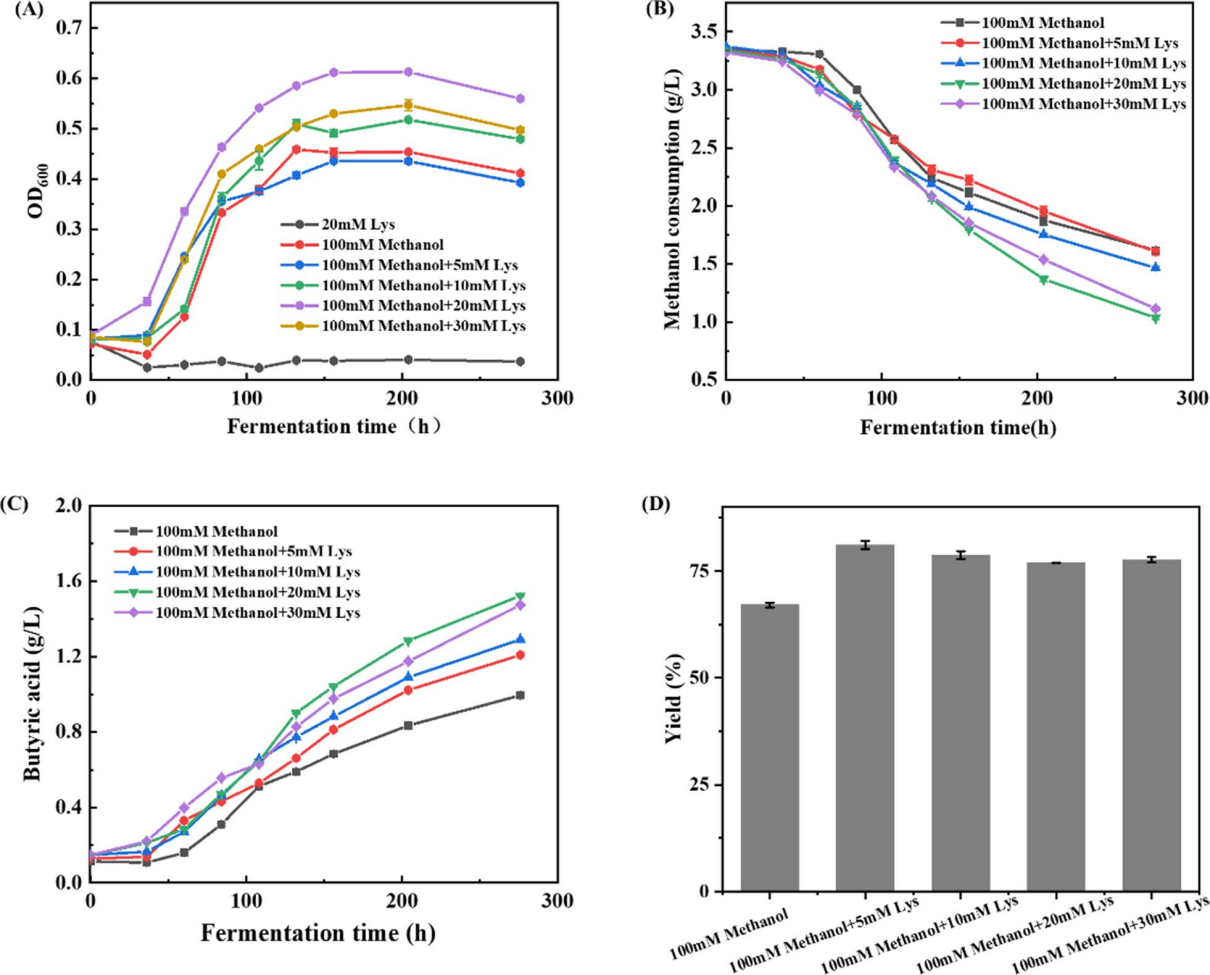
The effect of lysine supplementation on the methanol utilization and butyric acid production of *B. methylotrophicum.* The strain *B. methylotrophicum* was cultivated in modified DSM135 medium supplemented with extra concentrations of lysine (0 mM, 5 mM, 10 mM, 20 mM and 30 mM) anaerobically. The growth profiles (**A**), the methanol consumption (**B**), butyric acid production (**C**), and butyric acid yield (**D**) were determined

As high lysine level was supposed to be beneficial for improving methanol assimilation of *B. methylotrophicum*, the effect of different concentrations of lysine was further evaluated. The results are shown in Fig. 3. The lysine concentration ranged from 5 to 30 mM. When a small amount of 5 mM lysine was supplemented, the cell growth and methanol consumption of *B. methylotrophicum* were slightly affected (Fig. 3a, b). With a higher lysine concentration from 10 to 30 mM, the cell growth, methanol consumption and butyric acid production of *B. methylotrophicum* were significantly improved (Fig. 3a–c), further confirming our conclusion that increasing the availability of lysine level could enhance methanol assimilation of *B. methylotrophicum*. At the condition of 20 mM lysine, the cell growth, methanol consumption and butyric acid production of *B. methylotrophicum* reached the maximum level, which were increased by 38.44%, 21.52%, and 53.07%, respectively (Fig. 3a–c). In addition, we found that the addition of lysine could also improve the butyric acid yield from methanol (Fig. 3d), while the production of acetic acid was decreased (Additional file 1: Fig. S2). In acetogenic bacteria, several amino acids have been reported to be oxidized and degraded to support cell growth. For example, alanine could serve as growth substrate for *Sporomusa aerivorans* (*S. aerivorans*) *and A. woodii* [24, 25], and *E. limosum* was able to use isoleucine and valine as growth substrate [26]. We also evaluated whether lysine could be used as a growth substrate for *B. methylotrophicum* and therefore stimulated growth on methanol. As the result shows in Fig. 3a, in the cultivation medium containing 3 g/L yeast extract, methanol was removed and 20 mM lysine was supplemented to determine the effect on cell growth. The results showed that the cells of *B. methylotrophicum* could not grow when methanol was removed in presence of lysine, and none of lysine consumption was observed (Fig. 3a and Additional file 1: Fig. S3). Under the condition of 100 mM methanol supplemented with different lysine concentration, lysine was also barely consumed with methanol consumption and cell growth (Additional file 1: Fig. S3). These results indicated that lysine was not served as a growth co-substrate to improve methanol assimilation in *B. methylotrophicum.*

### Transcriptional analysis revealed that up-regulated expression of ABC transporters was triggered by lysine addition

**Fig. 4:**
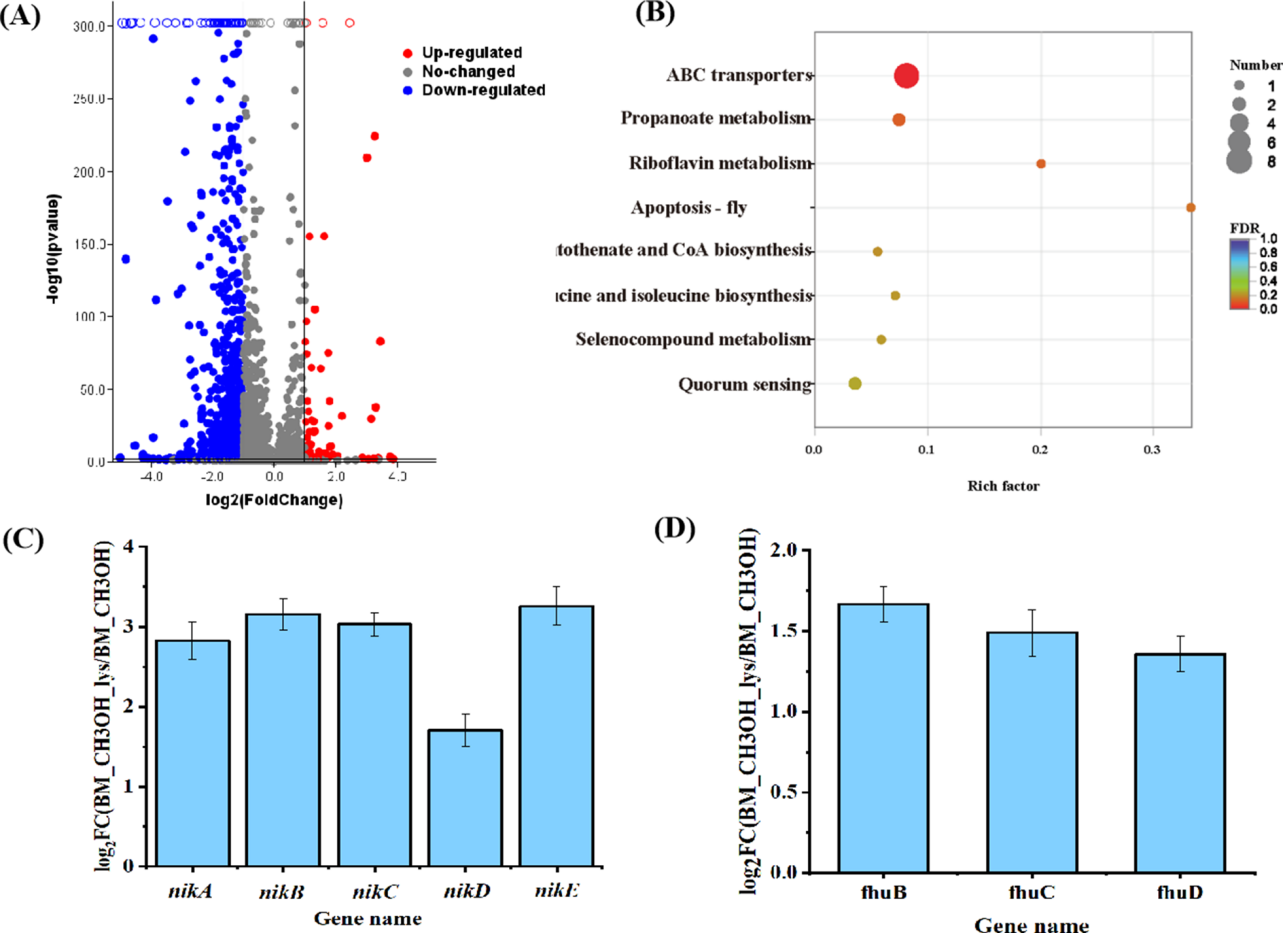
The transcriptional response to *B. methylotrophicum* to lysine addition*.* **A** Volcano map of differential genes in cells cultivated with and without extra lysine addition. Significantly differentially expressed genes are represented by red dots (up-regulated) and blue dots (down-regulated). The abscissa represents the fold change of gene expression in different samples, log_2_(Fold Change) > 1; the ordinate represents the statistical significance of the difference in gene expression, padjust < 0.05. **B** Pathway enrichment analysis of the significantly up-regulated genes with the corrected *p*-value < 0.05. **C** The transcriptional response of genes in NikABCDE tansporter system to lysine addition in *B. methylotrophicum.* **D** The transcriptional response of genes in FhuBCD transporter system to lysine addition in *B. methylotrophicum*

To elucidate the potential mechanism of lysine in regulating methanol assimilation of *B. methylotrophicum*, the transcriptional response to lysine addition was determined. Through the RNA-seq experiments, differentially expressed gene (DEG) analysis identified 58 up-regulated and 920 down-regulated genes (Fig. 4a). The significantly changed genes are illustrated in Additional file 1: Tables S2 and S3. KEGG pathway analysis was subsequently conducted to identify the pathways for these DEGs. With the corrected *p*-value < 0.05, the up-regulated genes were significantly enriched in 8 pathways, while the down-regulated genes were enriched in 7 pathways (Fig. 4b and Additional file 1: Fig. S4). In the enriched pathways for up-regulated genes, ABC transporters represented the most significant one (Fig. 4a). In the enriched pathways for down-regulated genes, the pathways involved in ribosome, fatty acid synthesis, phosphotransferase system, fructose and mannose metabolism and lysine biosynthesis changed most significantly (Additional file 1: Fig. S4).

Here, the genes in the up-regulated pathways attracted our interested. Eight significant up-regulated ABC transporters including *NikA*, *NikB*, *NikC*, *NikD*, *NikE*, *FhuB*, *FhuC*, and *FhuD* was clustered into two classes. The first cluster including genes of *NikA*, *NikB*, *NikC*, *NikD* and *NikE* was defined as the nickel transport system [27], while another gene cluster of *FhuB*, *FhuC*, and *FhuD* was defined as an iron complex transport system involved in the uptake of siderophores, heme and cobalamin (vitamin B12) [28]. In *B. methylotrophicum,* methanol was assimilated to acetyl-CoA with CO_2_ as an electron acceptor through the methyltransferase system along with the carbonyl branch of the WLP pathway [16]. In the methyltransferase system, the corrinoid-dependent methyltransferase (MtaB) first transfers the methyl group of methanol to a corrinoid protein (MtaC), and then methyltetrahydrofolate-methyltransferase (MtaA) transfers the methyl group from methyl-MtaC to tetrahydrofolate (THF), where cobalamin is an important cofactor for the activity of MtaB and MtaC [29], up-regulation of the FhuBCD transporter may enable MtaB and MtaC catalytic activity, thereby affecting methanol assimilation in *B. methylotrophicum*. In the carbonyl branch of the WLP pathway, both carbon monoxide dehydrogenase (CODH) and acetyl-CoA synthase (ACS) are nickel-containing enzymes [30], up-regulation of the NikABCDE transporter may enhance the uptake of nickel and affect the catalytic activity of CODH and ACS. The gene expression of *NikA*, *NikB*, *NikC*, *NikD*, and *NikE* was significantly up-regulated by lysine addition with fold changes of 7.9, 9.9, 8.8, 3.6, 10.8, respectively (Fig. 4c), and the expression of *FhuB*, *FhuC*, and *FhuD* was increased by 3.3-fold, 3.0-fold, and 2.7-fold, respectively (Fig. 4d). To further demonstrate whether lysine stimulated the up-regulation of NikABCDE and FhuBCD transporters, we carried out RT-qPCR experiments. Following the results shown in Additional file 1: Fig. S5, the relative expression level of NikABCDE and FhuBCD transporters was significantly up-regulated by 2.19 and 1.98 times, respectively, when 20 mM lysine was added into the methanol medium. We thus speculated that the up-regulated cofactor uptake system might be involved in the improved methanol metabolism triggered by lysine in *B. methylotrophicum*.

### Overexpression of *NikABCDE* or *FhuBCD* improved methanol assimilation of* B. methylotrophicum*

**Fig. 5:**
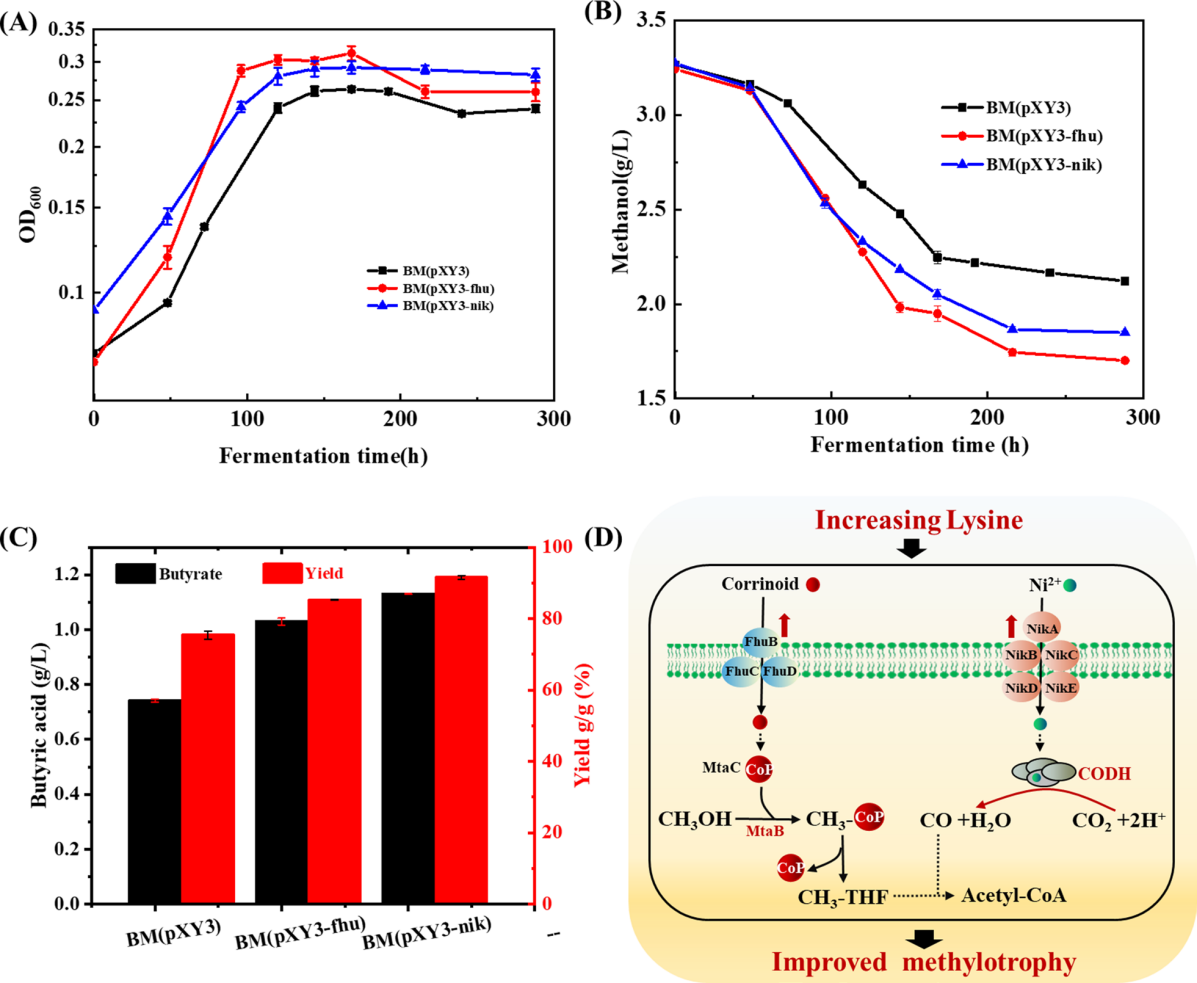
The overexpression of *NikABCDE* or *FhuBCD* transporter system confers the improved methylotrophy for *B. methylotrophicum.* **A** The effects of overexpressing *NikABCDE* or *FhuBCD* operon on the growth of *B. methylotrophicum.* **B** The effects of overexpressing *NikABCDE* or *FhuBCD* operon on the methanol consumption of *B. methylotrophicum.* **C** The effects of overexpressing *NikABCDE* or *FhuBCD* operon on the butyric acid of *B. methylotrophicum.* **D** The schematic depicting the potential mechanism of lysine regulation in methylotrophy for *B. methylotrophicum*

To further identify whether the up-regulation of these two-transport system was responsible for the improved methanol utilization of *B. methylotrophicum*, *NikABCDE* or *FhuBCD* was engineered to evaluate their effects on methanol metabolism of *B. methylotrophicum*. As shown in Fig. 5a, the overexpression of *FhuBCD* or *NikABCDE* resulted in a significant improvement in the methylotrophic phenotype over the empty plasmid control. With methanol as the sole carbon source, the specific growth rate of *NikABCDE* overexpressing strain reached 0.01393 h^−1^, 1.4-fold higher than the empty plasmid control, and the overexpression of *FhuBCD* increased the specific growth rate to 0.01641 h^−1^, 1.2-fold higher than the control (Fig. 5a). Higher final biomass titer was also achieved by the overexpression of *FhuBCD* or *NikABCDE* (Fig. 5a). At the meantime, the methanol consumption rate of the recombinant *B. methylotrophicum/*pXY1-*FhuBCD* and *B. methylotrophicum/*pXY1-*NikABCDE* increased by 24.5% and 34.7%, respectively (Fig. 5b). The titer and yield of butyric acid from the methanol were also significantly improved when these two systems were overexpressed (Fig. 5c). The butyric acid titer in the recombinant *B. methylotrophicum/*pXY1-*FhuBCD* and *B. methylotrophicum/*pXY1-*NikABCDE* was increased by 38.9% and 52.5%, respectively, and the butyric acid yield in the recombinant *B. methylotrophicum/*pXY1-*FhuBCD* and *B. methylotrophicum/*pXY1-*NikABCDE* was increased by 9.9% and 16.1%, respectively. Based on these results, we confirmed that the overexpression of *NikABCDE* or *FhuBCD* could improve methylotrophy of *B. methylotrophicum* (Fig. 5c). We thus proposed a possible regulatory mechanism that increasing lysine level triggered the expression of *NikABCDE* and *FhuBCD* transport system responsible for improved methanol utilization in *B. methylotrophicum* (Fig. 5d).

### The engineering of lysine synthetic pathway enhanced methanol utilization and butyric acid production in *B. methylotrophicum*

**Fig. 6:**
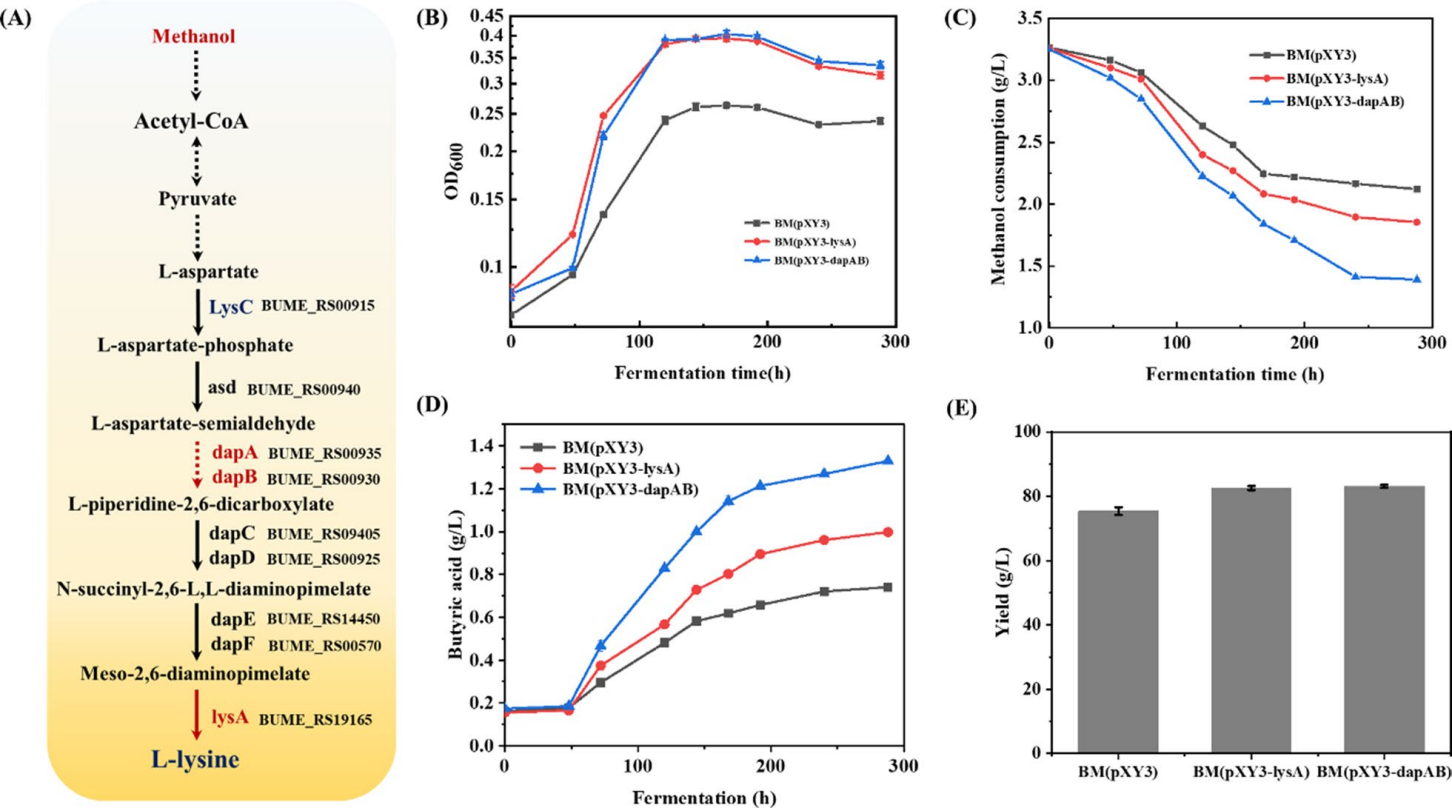
The overexpression of de novo lysine synthetic pathway enhances methanol utilization and butyric acid production for *B. methylotrophicum.* **A** The lysine synthetic pathway in *B. methylotrophicum*. **B** The effects of overexpressing *lysA* or *dapAB* operon on the growth of *B. methylotrophicum.* **C** The effects of overexpressing *lysA* or *dapAB* operon on the methanol consumption of *B. methylotrophicum.* **D** The effects of overexpressing *lysA* or *dapAB* operon on butyric acid production of *B. methylotrophicum.* **E** The effects of overexpressing *lysA* or *dapAB* operon on butyric acid yield of *B. methylotrophicum*

As exogenous lysine addition could improve methanol utilization of *B. methylotrophicum*, we hypothesized that increasing lysine synthesis through the genetic modification of the de novo lysine synthetic pathway was able to improve methanol assimilation. The lysine biosynthetic pathway in *B. methylotrophicum* was identified to be similar with that in *E. coli,* where L-aspartate was converted to generate L-lysine by the catalysis of *lysC*, *asd*, *dapA*, *dapB*, *dapC*, *dapD*, *dapE*, *dapF*, and *lysA* (Fig. 6a)*.* The overexpression of *lysA*, *dapA*, or *dapB* has been confirmed to be an efficient approach for enhancing flux through the lysine synthetic pathway [31–34]. Here, two plasmids of pXY1-P_thl_-*dapA*-*dapB* for the overexpression of *dapA* and *dapB*, and pXY1-P_thl_-*lysA* for the overexpression of *lysA* were constructed and electro-transformed into *B. methylotrophicum*. As the results illustrated in Fig. 6b, both the overexpression of *lysA* or co-overexpression of *dapA* and *dapB* could significantly enhance the methylotrophic phenotype of *B. methylotrophicum*. The biomass concentration and methanol consumption of *B. methylotrophicum/*pXY1-P_thl_-*lysA* was increased by 38.8%, and 23%, respectively (Fig. 6b, c). The recombinant strain *B. methylotrophicum/*pXY1-P_thl_-*dapA*-*dapB* exhibited the best growth advantage in methanol medium, in which the final biomass concentration was increased by 51%, and the specific growth rate reached 0.0259 h^−1^ (Fig. 6b). The methanol consumption of *B. methylotrophicum/*pXY1-P_thl_-*dapA*-*dapB* was increased by 63.2% (Fig. 6c). In addition, the enhancement of lysine could also improve butyric acid production titer and yield from methanol (Fig. 6d, e). The overexpression of *lysA* increased the butyric acid production by 33.8% with a final titer of 0.99 g/L, while the accumulation of acetic acid was significantly decreased (Additional file 1: Fig. S6). For the engineered *B. methylotrophicum/*pXY1-P_*thl*_-*dapA*-*dapB*, a titer of 1.33 g/L butyric acid was achieved with a yield of 83.1%, which was increased by 79.7% and 10.4% compared to that in the control strain, respectively (Fig. 6c, d). These results confirmed that increasing the flux through the lysine biosynthetic pathway could efficiently improve methanol utilization and butyric acid production of *B. methylotrophicum*, further identifying that lysine is an important target for the regulation of methanol utilization in *B. methylotrophicum.*

**Fig. 7:**
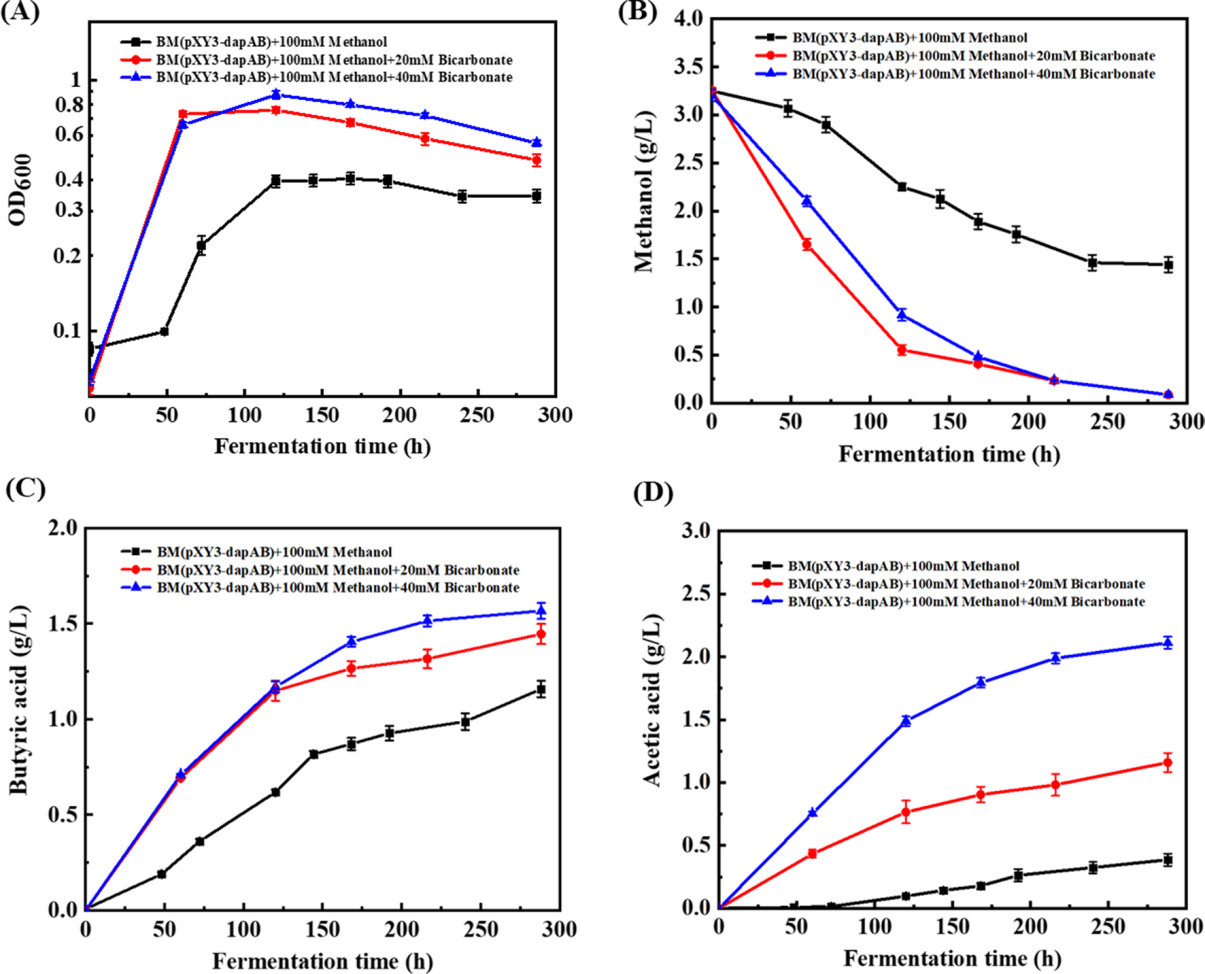
The presence of CO_2_ to improve methanol utilization and butyric acid production of *B. methylotrophicum*. The growth profiles (**A**), methanol consumption (**B**), butyric acid production (**C**), and acetic acid production (**D**) of *B. methylotrophicum* under 100 mM methanol condition with the addition of 0 mM, 20 mM, and 40 mM sodium bicarbonate

**Fig. 8:**
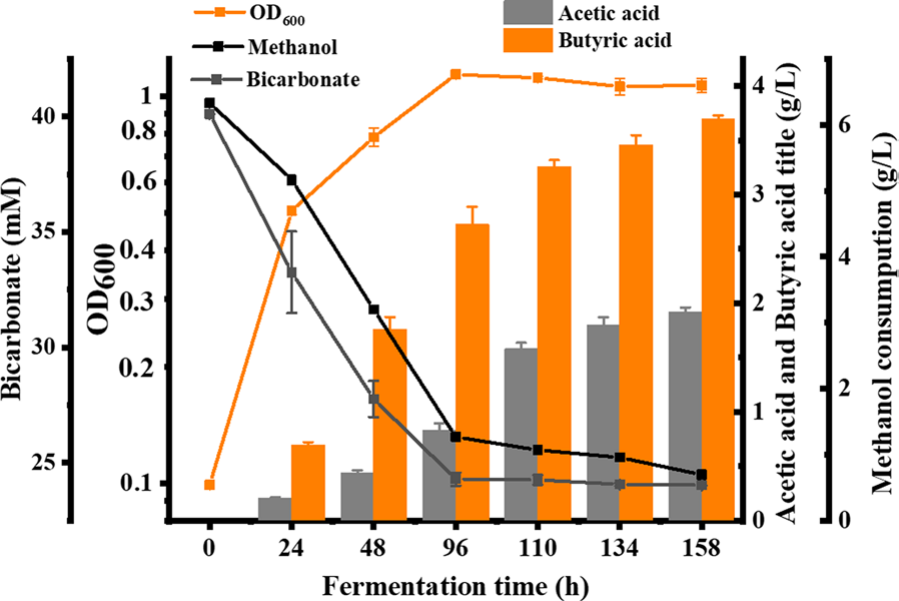
The fermentation of the engineered *B. methylotrophicum*/pXY1-P_thl_-*dapA*-*dapB* in CSL medium supplemented with 200 mM methanol and 40 mM bicarbonate

The presence of CO_2_ as an electron acceptor could affect methanol consumption and product distribution in *B. methylotrophicum.* Different concentrations of bicarbonate were therefore supplemented and the cell growth on methanol was largely improved (Fig. 7a). The percentage of methanol consumption was increased to 97% in conditions of 20 mM or 40 mM bicarbonate from 55% in conditions of 0 mM bicarbonate (Fig. 7b). The titer of butyric acid reached 1.45 g/L when the methanol to bicarbonate ratio was 100 mM:20 mM (Fig. 7c). With the further increase of bicarbonate concentration to 40 mM, butyric acid titer was just increased to 1.6 g/L, and meanwhile large amount of acetic acid (2.0 g/L) was produced (Fig. 7d). To further improve the butyric acid production of the engineered *B. methylotrophicum/*pXY1-P_thl_-*dapA*-*dapB* from methanol, the fermentation was performed in CSL medium. As shown in Fig. 8, under the condition of 200 mM methanol and 40 mM bicarbonate, the cell grew into the stationary phase after the fermentation 96 h. With the prolonged fermentation time to 158 h, the production of butyric acid reached 3.69 g/L, while 1.92 g/L acetic acid was accumulated. Finally, 5.7 g/L methanol and 15.97 mM bicarbonate were totally consumed, and the yield of butyric acid from methanol could reach 76.3%.

## Discussion

Methanol is an abundant and attractive fermentation substrate as an alternative to sugars with the advantage of high electron and energy content. Recent attempts to develop methylotrophic cell factories have been made by using native or synthetic methylotrophs [35, 36]. Nowadays, most of studies have focused on the aerobic methylotrophs. For example, through the engineering of aerobic methylotrophic *P. pastoris*, Zhou et al. enable high-level production of free fatty acid or fatty alcohols from sole methanol [37, 38]. Meanwhile, anaerobic methanol utilization is also an interesting area as anaerobic conditions are also desirable for production of many metabolites, such as organic acids or alcohols [39]. *B. methylotrophicum* is one of representative anaerobic methylotrophic bacteria. Specially, CO_2_ could be used as the sole electron acceptor during methanol assimilation, making *B. methylotrophicum* as an ideal platform for C1-compound bioconversion. Compared to the aerobic methylotrophs, anaerobic methylotrophs are relatively slow growing and achieve relatively low cell densities. Rare knowledge on the understanding of regulating methylotrophic ability of anaerobic methylotrophic bacteria also limited their rational engineering for efficient C1-compound bioconversion.

Nitrogen source, one of nutrient component essential for cell growth, has been shown to affect methanol metabolism ability in various native methylotrophs, such as *P. pastoris* [21], *Methylomicrobium album* [40] or *Methylocystis sp* [22]*.* In a previous study, the use of yeast extract could also improve methanol utilization in a synthetic methylotrophic *E. coli*, Subsequent work determined that this stimulatory effect was responsible threonine [41]. In another study, it was found that triggering the stringent response to increase the synthesis of several amino acids could also improve cell growth on methanol [42], indicating the important role of amino acid on methanol metabolism in methylotrophs. Here, we found that the use of corn steep liquor significantly improved methylotrophic ability of *B. methylotrophicum*. Further investigation revealed that high level of lysine contributed to the improved methylotrophic ability of *B. methylotrophicum.* Several amino acids could be oxidized to support cell growth of methylotrophic-acetogenic bacterium. For example, *E. limosum* is able to use isoleucine and valine as substrates [26], and alanine could serve as substrate for *S. aerivorans and A. woodii* [24, 25]. However, lysine could not support cell growth of *B. methylotrophicum* as observed in our results (Fig. 3a), indicating that the improved methylotrophic ability by lysine addition was not due to the extra presence of growth substrate.

The transcriptional response of *B. methylotrophicum* to lysine addition revealed that eight ABC transporters clustered into two classes of NikABCDE and FhuBCD are specifically up-regulated. NikABCDE transporter system is the main importer of nickel in microorganisms [43, 44]. Nickel is an essential micronutrient for a wide variety of microorganisms, and involves in numerous of cellular processes, serving as a cofactor for nickel-dependent enzymes [27, 45]. In the methylotrophic-acetogenic bacterium, nickel is essential metallocofactor for the activity of CODH and ACS, which are responsible for CO_2_ reduction and acetyl-CoA synthesis in methanol assimilation pathway [46]. The up-regulation of NikABCDE by lysine addition in *B. methylotrophicum* might enhance the uptake of nickel, increase the catalytic activity of CODH and ACS, and thus impact methylotrophy. Another FhuBCD transporter system up-regulated by lysine addition was involved in the uptake of cobalamin, an essential cofactor for the activity of *MtaB* and *MtaC*, which catalyzes the initial methyltransfer step in methanol assimilation pathway of *B. methylotrophicum.* The improved methylotrophy through the overexpression of *NikABCDE* or *FhuBCD* further suggested the potential mechanism by which lysine addition could regulate methylotrophic ability of *B. methylotrophicum* (Fig. 5).

Based on the results in this study, we confirm that lysine plays an important role in regulating methylotrophy activity of *B. methylotrophicum,* which referred to ABC transporter involved in the uptake of essential cofactors for enzymes in methanol assimilation pathway. Amino acid metabolism has been reported to be an important factor in regulating methanol metabolism in several methylotrophs [41, 42]. When we performed amino acid addition experiments, we also found that the addition of Gly, Val and Ala could show a moderate increase in final biomass. The different amino acids might have a synergistic effect on methanol utilization, which would be further investigated in our following works. To our knowledge, this is the first report to uncover the regulatory role of amino acid in methylotrophy activity of methylotrophic-acetogenic bacterium. Based the proposed targets, *B. methylotrophicum* was further engineered by enhancing the endogenous lysine synthesis (Fig. 8), 3.69 g/L butyric acid was finally produced from methanol, which was the highest titer reported so far by *B. methylotrophicum* with methanol and bicarbonate as the sole carbon source [19, 47, 48]. Our study not only identified new gene targets for understanding methanol metabolism mechanism in methylotrophic-acetogenic bacterium, but also constructed several engineered strains with improved methylotrophy, which could serve as customized host strains for methanol bioconversion to produce variate value-added chemicals.

## Conclusions

Here, we revealed a potential regulation mechanism for improved methanol metabolism in *B. methylotrophicum.* Higher level of lysine was identified to be responsible for enhanced methanol utilization of *B. methylotrophicum.* With the transcriptome analysis, we found that lysine could up-regulate cofactor transporters essential for enzymes of methanol assimilation. The regulation mechanism of lysine was further confirmed by improved methylotrophy of *B. methylotrophicum* when overexpressing *NikABCDE or FhuBCD* operon. Based on our proposed gene targets, the de novo synthetic pathway of lysine was enhanced*,* and the bioconversion of methanol to butyric acid was successfully improved*.*

## Materials and methods

### Microbial strains and growth conditions

All strains used in this work are listed in Table 1. *Escherichia coli* strains were cultivated at 37 °C in Luria–Bertani medium (tryptone 10 g/L, yeast extract 5 g/L, NaCl 10 g/L) supplemented with appropriate antibiotics at the following concentrations: 100 μg/mL spectinomycin (Spe), or 100 μg/mL ampicillin (Amp).

**Table 1:** Strains and plasmids used in this study

Strains or plasmids	Description of genotype	Source
*Strains*		
*E. coli* Trans T1	F¯φ80(*lacZ*)ΔM15Δ*lacX*74 *hsd*R(rK^−^, mK^+^) Δ*rec*A 1398 *end*A1 *ton*A	TransGen
*E. coli* Top10	F¯*mcr*A Δ (*mrr-hsd* RMS-*mcr*BC) φ80 *lacZ*ΔM15Δ lacX74 *rec*A *ara*Δ139(*ara-leu*)7697 *gal*U *gal*K *rps*L(Str^R^) *end*A1 *nup*G	TransGen
*B. methylotrophicum*	Wild type	ATCC
*B. methylotrophicum/*pXY1-*NikABCDE*	*B. methylotrophicum* overexpressing the operon of NikABCDE under the control of P_thl_	This study
*B. methylotrophicum/*pXY1-*FhuBCD*	*B. methylotrophicum* overexpressing the operon of *FhuBCD* under the control of P_thl_	This study
*B. methylotrophicum/*pXY1-LysA	*B. methylotrophicum* overexpressing the gene of *LysA* under the control of P_thl_	This study
*B. methylotrophicum/*pXY1-DapAB	*B. methylotrophicum* overexpressing the gene of *DapA and DapB* under the control of P_thl_	This study
*Plasmids*		
pMCljS	pACYC184, methyltransferase gene of *C. ljungdahlii* (CLJU c03310), Spe^R^	[16]
pXY1	pCB102 ORI, ColE1 origin; Amp^R^, Em^R^	[16]
pXY1-P_*thl*_-*NikABCDE*	Derived from pXY1, with the operon of *nikA*, *nikB, nikC, nikD* and *nikE* overexpression	This study
pXY1-P_*thl*_-*FhuBCD*	Derived from pXY1, with the operon of *FhuB*, *FhuC* and *FhuD* overexpression	This study
pXY1-LysA	Derived from pXY1, with the gene of *LysA* overexpression	This study
pXY1-DapAB	Derived from pXY1, with the gene of *DapA* and *DapB* overexpression	This study

Spe^R^, spectinomycin resistance; Amp^R^, ampicillin resistance; Em^R^, erythromycin resistance; pCB102 ORI, Gram-positive origin of replication from *Clostridium butyricum*

*Butyribacterium methylotrophicum* ATCC 33266 was cultivated anaerobically at 37 °C in modified DSM 135 medium described previously in an anaerobic chamber (AW500TG, Electro-Tech, Co., Ltd., UK) [16]. 30 μg/mL of erythromycin (Em) was supplemented when recombinant *B. methylotrophicum* strains were cultivated. The certain concentration of methanol (100 mM-200 mM) was added as the carbon source. To determine the effect of nitrogen source on methanol utilization, 2.64 g/L peptone, 4.13 g/L beef extract, 8.25 g/L corn milk, 0.17 g/L urea, 0.17 g/L ammonium sulfate, or 0.34 g/L ammonium chloride, which contained the equal nitrogen content as 3 g/L yeast extract, was supplemented to replace the yeast extract in the modified DSM 135 medium. To determine the effect of amino acid on methanol utilization, 5 mM of glycine (Gly), alanine (Ala), lysine (Lys), asparagine (Asn), threonine (Thr), glutamate (Glu), aspartate (Asp), valine (Val), isoleucine (Ile), serine (Ser), tyrosine (Tyr), proline (Pro), or phenylalanine (Phe) were added into the modified DSM 135 medium with 100 mM or 200 mM methanol as the sole carbon source. To evaluate the effect of lysine on methanol metabolism, different concentrations of sodium bicarbonate (5 mM, 10 mM, 20 mM, and 30 mM) were added into the modified DSM 135 medium with a fixed methanol concentration of 100 mM.

### Analysis of the amino acid composition

Culture broth of *B. methylotrophicum* in medium supplemented with corn steep liquor (CSL) or yeast extract (YE) was sampled at 0 h, 36 h (middle exponential phase), 60 h (later exponential phase), and 84 h (stationary phase), respectively. After the centrifugation at 13,000 g for 10 min, the supernatant was taken and filtered. 200 μL of supernatant was lyophilized under low temperature under low temperature (− 60 °C). The dried samples were derivatized at 40 °C for 160 min with 50 μL methoxamine hydrochloride (20 mg/mL in pyridine) and 80 μL N-methyl-N-(trimethylsilyl) trifluoroacetamide (MSTFA). Derivatized samples were subsequently analyzed using a TRACE 1310 GC system (Thermo Fisher Scientific, USA) combined with ISQ 7000 MS system (Thermo Fisher Scientific, USA), which was equipped with a fused-silica capillary column (30 m × 0.25 mm i.d., 0.25 μm DB-5MS stationary phase, J&W Scientific, Folsom, CA). Four replicates were performed for each sample.

### Transcriptome analysis

Cells of *B. methylotrophicum* cultivated in modified DSM 135 medium with/without 20 mM of lysine was sampled at the middle exponential phase, respectively, for transcriptome analysis. Total RNA was extracted using TRIzol^®^ Reagent according to the manufacturer’s instructions (Invitrogen). The genomic DNA was removed using DNase I (TaKaRa). RNA quality was then determined with a 2100 Bioanalyzer (Agilent) and quantified with an ND-2000 (NanoDrop Technologies). An RNA-seq transcriptome library was prepared following instructions in the TruSeq™ RNA sample preparation Kit from Illumina (San Diego, CA). The data generated from the Illumina platform were used for bioinformatics analysis. All of the analyses were performed using the free online Majorbio Cloud Platform (www.majorbio.com) from Shanghai Majorbio Bio-pharm Technology Co., Ltd.

### RNA isolation and reverse transcription quantitative PCR

*B. methylotrophicum* was cultivated in modified DSM 135 medium with the addition of 100 mM methanol or 100 mM methanol and 20 mM lysine. Cells at the exponential growth stage were harvested by centrifugation (4000 × *g*, 30 min), washed three times using ultra-pure water, and immediately frozen in liquid nitrogen. The total RNA was purified with the RNAprep Pure Cell/Bacteria kit (Tiangen Biotech, China), and the cDNA was obtained by reverse transcription of the total RNA and used as the template for quantitative real-time PCR (qPCR).

The qPCR was performed with a Light Cycler Instrument (Thermo Fisher Scientific, USA). The PCR reaction was performed with SYBR Premix Ex Taq (Takara) and the primers for *NikABCDE* and *NikABCDE* are listed in Additional file 1: Table S1.

### Plasmid construction and transformation

All plasmids used in this work are shown in Table 1. The genes *lysA, dapA,* and *dapB* were amplified from the genome of *B. methylotrophicum* using the primer pairs of lysA-F/lysA-R and dapAB-F/dapAB-R, respectively, and then ligated into the plasmid pXY3 (linearized by the *BamHI* and *XbaI*) through the homologous recombination obtaining the plasmid pXY3-P_thl_-*lysA* and pXY3-P_thl_-*dapAB*. To overexpress the genes related to the ion channels, the operon of *NikABCDE* and *FhuBCD* were amplified from the genome of *B. methylotrophicum* and ligated into the plasmid pXY3 (linearized by the *BamHI* and *XbaI*) by homologous recombination, respectively. The primers used in the recombinant plasmid construction are shown in Additional file 1: Table S1. Before transformation into *B. methylotrophicum*, plasmids were transformed into *E. coli* Top10 containing methylation plasmid pMCljS for in vivo methylation. Subsequently, the methylated plasmids were extracted from *E. coli* and electro-transformed into *B. methylotrophicum.*

### Analytical techniques

During the fermentation, the cells were harvested by centrifugation at 12,000 rpm for 5 min and were resuspended with deionized water for the measurement of optical density at 600 nm with a UV–VIS spectrophotometer. The liquid supernatant of fermentation broth was used to measure the concentration of methanol, acetate and butyric acid by a high-performance liquid chromatography equipped with a refractive index detector and a column (Bio-Rad HPX-87H). The column temperature was 60 °C. The mobile phase was 8 mM H_2_SO_4_ solution with a flow rate was 0.6 mL/min. The concentration of lysine was analyzed by an SBA-40C biosensor analyzer (Shandong Province Academy of Sciences, China).

A SP-7890Plus gas chromatograph (Shandong Lunan Ruihong, Chinese) equipped with a TCD detector was used to calculate the net CO_2_ consumption rate (*n* = 3). To calculate the bicarbonate consumption rate by *B. methylotrophicum*, the headspace CO_2_ concentration (V/V) was measured by collecting headspace gas in different time periods. Then using calculation Eq. (1) to convert CO_2_ concentration (mM). The concentration conversion of sodium bicarbonate and CO_2_ was carried out as in Eq. 2:

1 $$ C_{{{\text{mM,CO}}_{{2}} }} = \frac{1000}{{22.4}}*C_{{v,{\text{CO}}_{2} }} *\frac{273*pa}{{\left( {273 + t} \right)*101.3}}, $$

2 $$ C_{{{\text{mM,NaHCO}}_{3} }} *V_{{{\text{NaHCO}}_{3} }} = C_{{{\text{mM,CO}}_{2} }} *V_{{{\text{CO}}_{2} }} . $$

## Supplementary Information

**Additional file 1. Fig. S1**: The changes of amino acids in YE medium (**A**) and CSL medium. **B** during the fermentation process of *B. methylotrophicum*. **Fig. S2**: The acetic acid production of *B. methylotrophicum* in medium supplemented with different concentrations of lysine. **Fig. S3**: The lysine consumption of *B. methylotrophicum* in medium supplemented with different concentrations of lysine. **Fig. S4**: Pathway enrichment analysis of the significantly down-regulated genes of *B. methylotrophicum* in response to lysine addition with the corrected *p*-value < 0.05. **Fig. S5**: Transcriptional levels of the NikABCDE and FhuBCD transporters in response to lysine in *B. methylotrophicum*. **Fig. S6**: The effects of overexpressing lysA or dapAB operon on acetic acid production of *B. methylotrophicum*. **Table S1**: The primers used in this study. **Table S2**: Significantly up-regulated genes of *B. methylotrophicum* in response to lysine addition. **Table S3**: Significantly down-regulated genes of *B. methylotrophicum* in response to lysine addition.

## Data Availability

The datasets used and analyzed during the current study are available from the corresponding author on reasonable request.

## Abbreviations

C1: One-carbon
CSL: Corn steep liquor
WLP: Wood–Ljungdahl pathway
Spe: Spectinomycin
Amp: Ampicillin
Em: Erythromycin
Gly: Glycine
Ala: Alanine
Lys: Lysine
Asn: Asparagine
Thr: Threonine
e Glu: Glutamate
Asp: Aspartate
Val: Valine
Ile: Isoleucine
Ser: Serine
Tyr: Tyrosine
Pro: Proline
Phe: Phenylalanine
YE: Yeast extract
DEG: Differentially expressed gene
MtaB: The corrinoid-dependent methyltransferase
MtaC: A corrinoid protein
MtaA: Methyltetrahydrofolate-methyltransferase
THF: Tetrahydrofolate
CODH: Carbon monoxide dehydrogenase
ACS: Acetyl-CoA synthase
