# Supplementary material for: Increasing lysine level improved methanol assimilation toward butyric acid production in Butyribacterium methylotrophicum
Source: Biotechnol Biofuels Bioprod. 2023 Jan 17;16:10. doi: 10.1186/s13068-023-02263-w (PMC9847067; doi:10.1186/s13068-023-02263-w)
Supplement: Supplementary file 1 — Additional file 1. Fig. S1: The changes of amino acids in YE medium (A) and CSL medium. B during the fermentation process of B. methylotrophicum. Fig. S2: The acetic acid production of B. methylotrophicum in medium supplemented with different concentrations of lysine. Fig. S3: The lysine consumption of B. methylotrophicum in medium supplemented with different concentrations of lysine. Fig. S4: Pathway enrichment analysis of the significantly down-regulated genes of B. methylotrophicum in response to lysine addition with the corrected p-value < 0.05. Fig. S5: Transcriptional levels of the NikABCDE and FhuBCD transporters in response to lysine in B. methylotrophicum. Fig. S6: The effects of overexpressing lysA or dapAB operon on acetic acid production of B. methylotrophicum. Table S1: The primers used in this study. Table S2: Significantly up-regulated genes of B. methylotrophicum in response to lysine addition. Table S3: Significantly down-regulated genes of B. methylotrophicum in response to lysine addition. [file 13068_2023_2263_MOESM1_ESM.docx]

# Original articles

# Increasing lysine level improved methanol assimilation toward butyric acid production in [*Butyribacterium methylotrophicum*](http://www.baidu.com/link?url=a08t7ZfLRpoPwIik17uVjRE3V-ht7i5jW_tFgbnBx5kjVx85Ai1twWP8CjUZGbW36uXW93jmoGQM65MFQ_SFjq)

**Jing Wang, Yang Liao, Jialun Qin, Chen Ma, Yuqi Jin, Xin Wang^*^, Kequan Chen, Pingkai Ouyang**

State Key Laboratory of Materials-Oriented Chemical Engineering, College of Biotechnology and Pharmaceutical Engineering, Nanjing Tech University, Nanjing 211816, Jiangsu, China

*Corresponding authors: Xin Wang

Email: xinwang1988@njtech.edu.cn


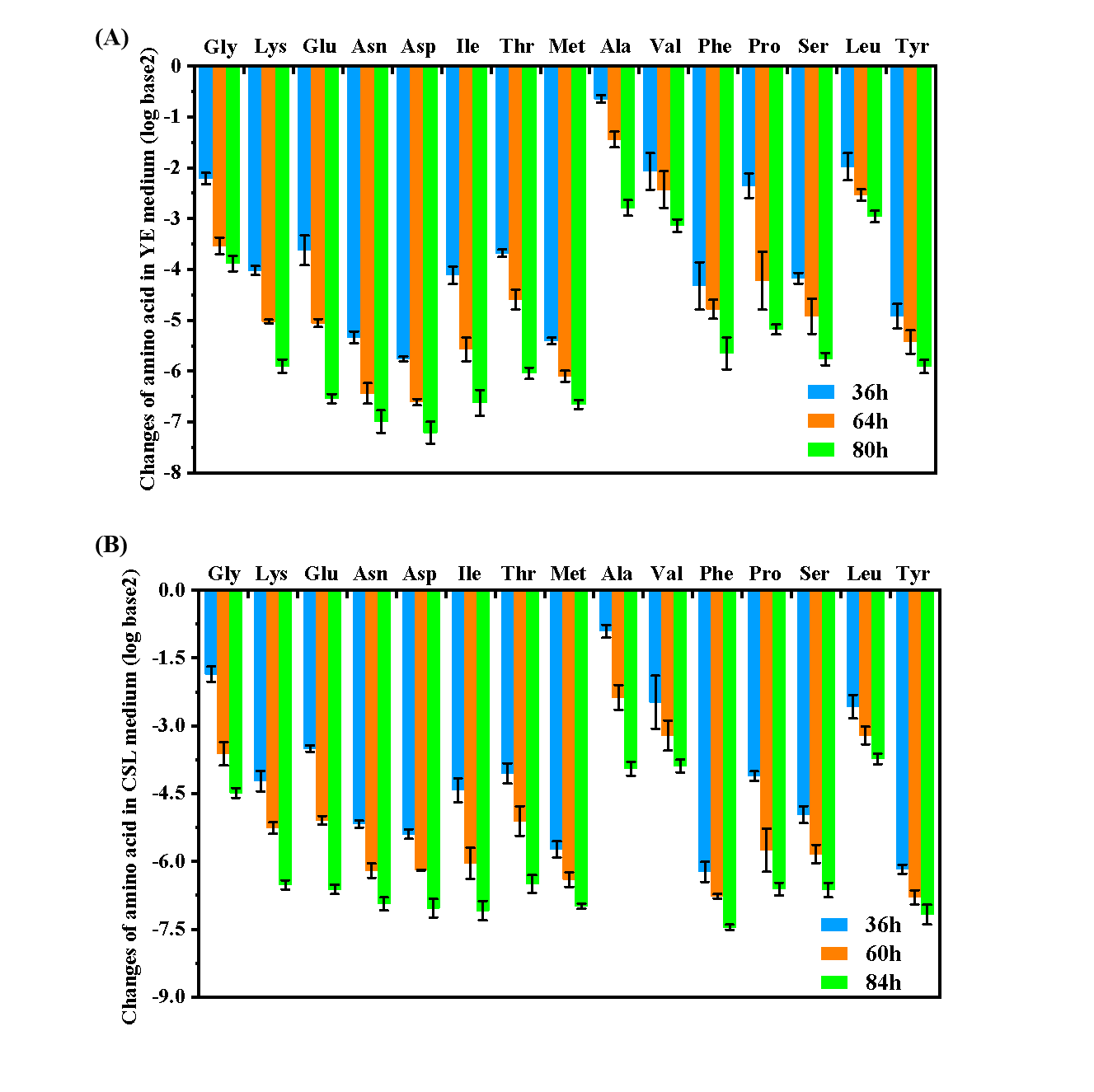


Figure S1 The changes of amino acids in YE medium (A) and CSL medium (B) during the fermentation process of [*B. methylotrophicum*](http://www.baidu.com/link?url=a08t7ZfLRpoPwIik17uVjRE3V-ht7i5jW_tFgbnBx5kjVx85Ai1twWP8CjUZGbW36uXW93jmoGQM65MFQ_SFjq)


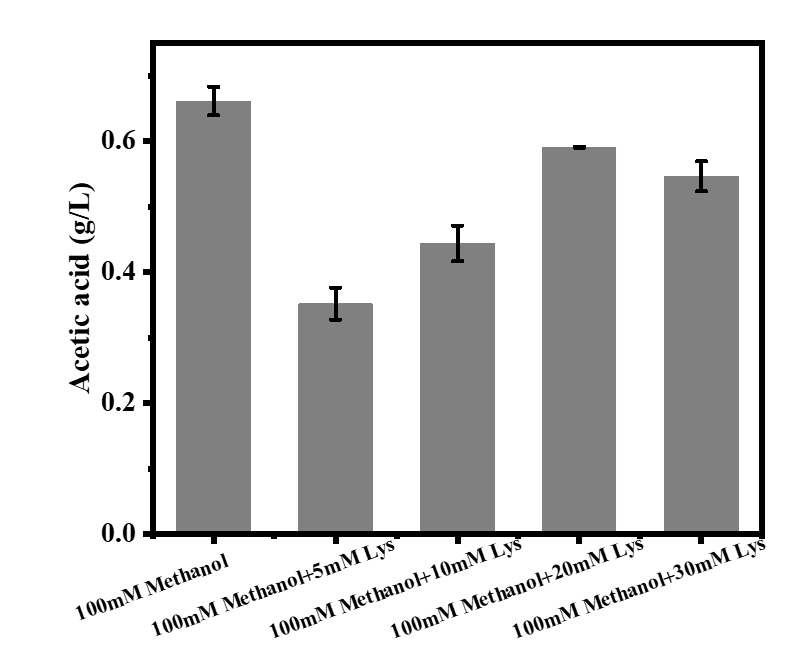


Figure S2 The acetic acid production of [*B. methylotrophicum*](http://www.baidu.com/link?url=a08t7ZfLRpoPwIik17uVjRE3V-ht7i5jW_tFgbnBx5kjVx85Ai1twWP8CjUZGbW36uXW93jmoGQM65MFQ_SFjq) in medium supplemented with different concentrations of lysine.


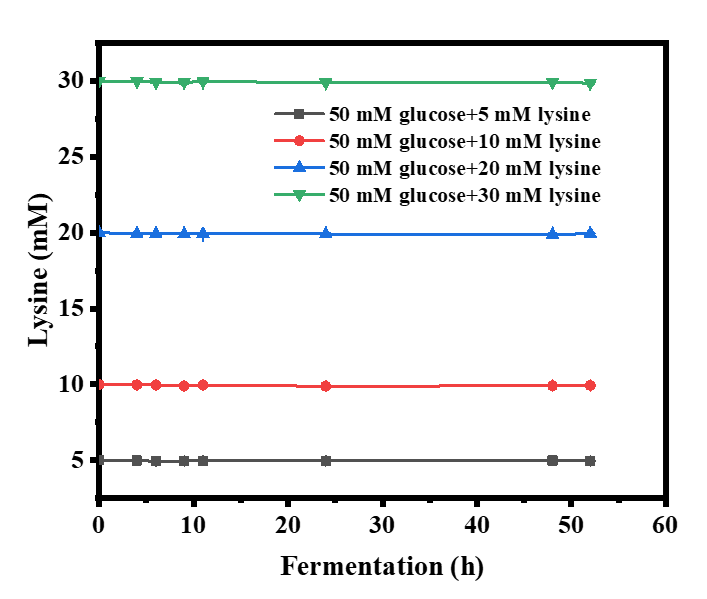


Figure S3 The lysine consumption of [*B. methylotrophicum*](http://www.baidu.com/link?url=a08t7ZfLRpoPwIik17uVjRE3V-ht7i5jW_tFgbnBx5kjVx85Ai1twWP8CjUZGbW36uXW93jmoGQM65MFQ_SFjq) in medium supplemented with different concentrations of lysine.


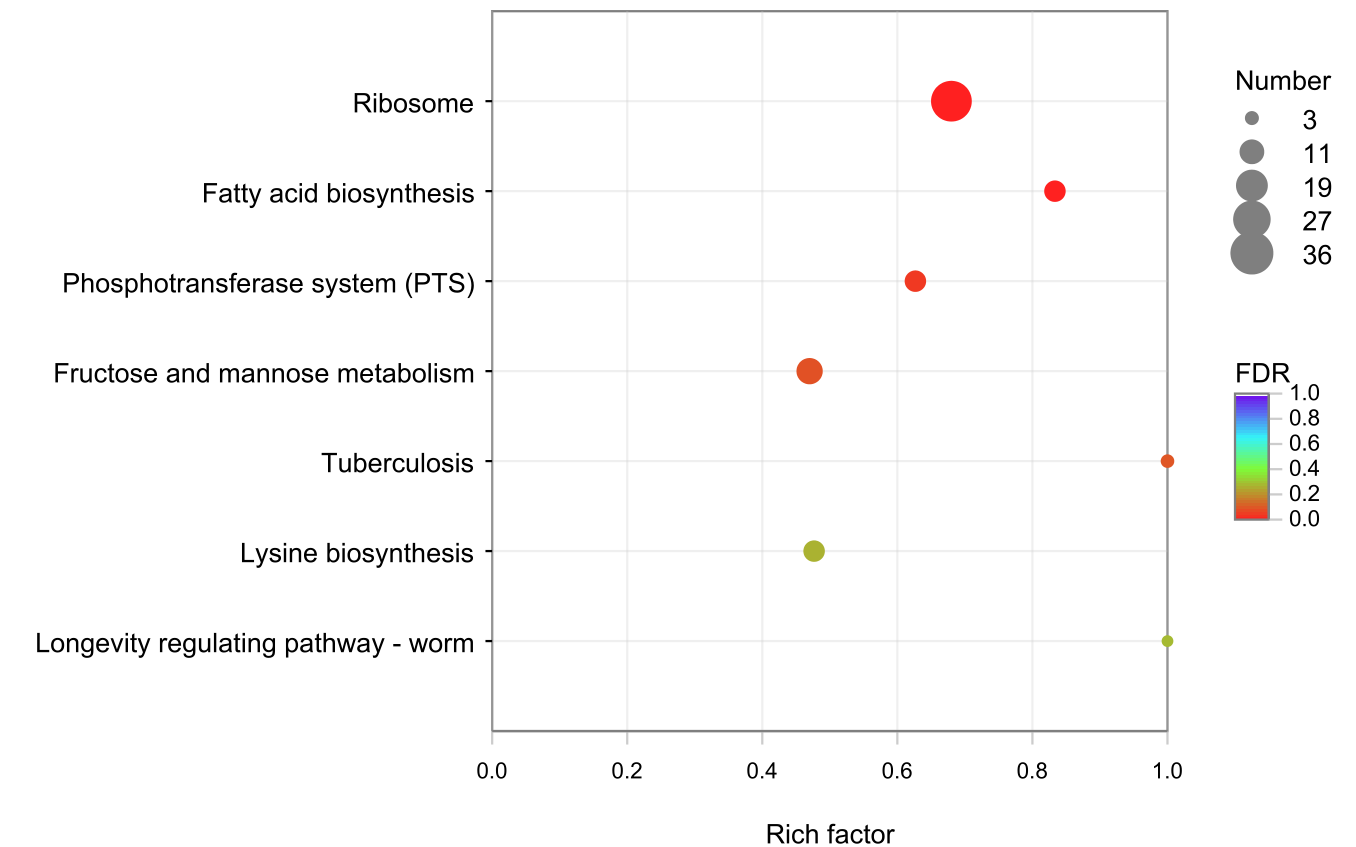


Figure S4 Pathway enrichment analysis of the significantly down-regulated genes of [*B. methylotrophicum*](http://www.baidu.com/link?url=a08t7ZfLRpoPwIik17uVjRE3V-ht7i5jW_tFgbnBx5kjVx85Ai1twWP8CjUZGbW36uXW93jmoGQM65MFQ_SFjq) in response to lysine addition with the corrected p-value <0.05.


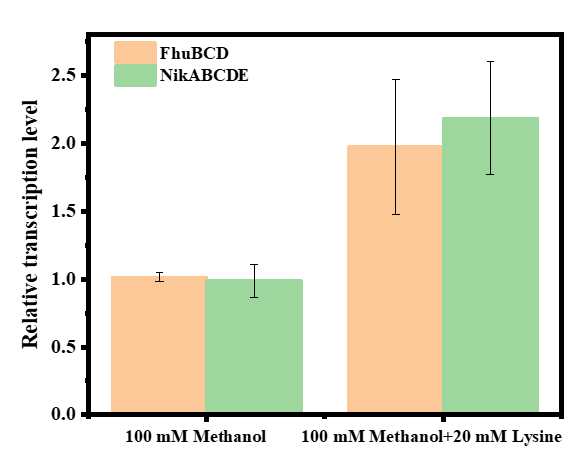


Figure S5 Transcriptional levels of the NikABCDE and FhuBCD transporters in response to lysine in [*B. methylotrophicum*](http://www.baidu.com/link?url=a08t7ZfLRpoPwIik17uVjRE3V-ht7i5jW_tFgbnBx5kjVx85Ai1twWP8CjUZGbW36uXW93jmoGQM65MFQ_SFjq) .


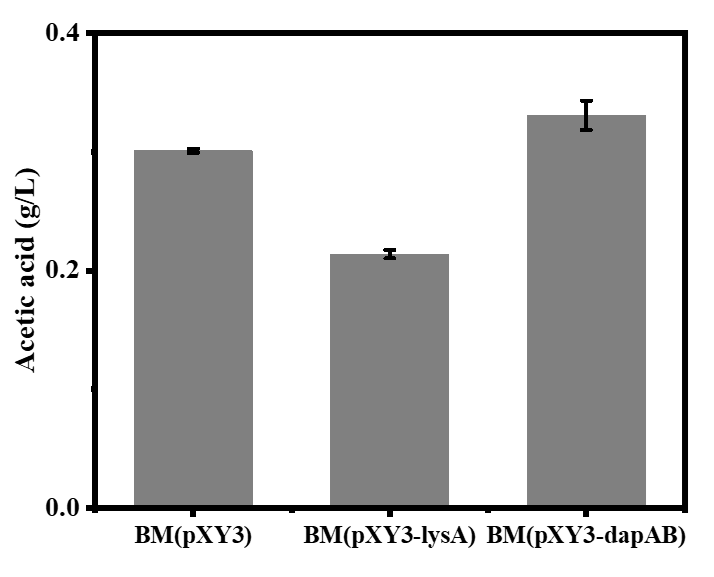


Figure S6 The effects of overexpressing *lysA* or *dapAB* operon on acetic acid production of [*B. methylotrophicum*](http://www.baidu.com/link?url=a08t7ZfLRpoPwIik17uVjRE3V-ht7i5jW_tFgbnBx5kjVx85Ai1twWP8CjUZGbW36uXW93jmoGQM65MFQ_SFjq).

**Table S1 The primers used in this study**

| **Primers** | **Sequences (5′–3′)** |
| --- | --- |
| fhuBCD-HR-F | AGGAGGTTAGTTAGAGGATCCATGAAAATGAAAAAGAGACTGGGTCAGG |
| fhuBCD-HR-R | ATTCTCGAGAGATCTTCTAGATTATTCTGGCACGACCATTTTATAGCCA |
| nikABCDE-HR-F | AGGAGGTTAGTTAGAGGATCCATGAAGCTTAAAAAAATCGCGGCG |
| nikABCDE-HR-R | ATTCTCGAGAGATCTTCTAGACTATACTTCAAAGGGGATTACGGATTCCAG |
| NikABCDEq-F | tcatcatactggtggtctcggt |
| NikABCDEq-R | ggtcataccgctggtaggca |
| FhuBCDq-F | aatttggtgggagatgtcctgg |
| FhuBCDq-R | cgcctcagtcccctcaatg |
| lysA-F | GTTAGTTAGAGGATCCATGACAGAAAATTTTGTATTTTCCGGTCATGAC |
| lysA-R | CGAGAGATCTTCTAGATTAGTCCTCTAGCCAGGAGGG |
| dapAB-F | GTTAGTTAGAGGATCCATGTCCACTATTTTTACAGGAAGCTGTG |
| dapAB-R | CGAGAGATCTTCTAGATTAATTGTCAATTAGCATTTCCATATTGTAGAGGCC |

**Table S2 Significantly up-regulated genes of** [***B. methylotrophicum***](http://www.baidu.com/link?url=a08t7ZfLRpoPwIik17uVjRE3V-ht7i5jW_tFgbnBx5kjVx85Ai1twWP8CjUZGbW36uXW93jmoGQM65MFQ_SFjq) **in response to lysine addition**

| Gene_id | Gene description | Fold Change | Log_2_ (FC) |
| --- | --- | --- | --- |
| BUME_RS12555 | hypothetical protein | 14.657 | 3.874 |

| BUME_RS18165 | riboflavin synthase | 13.61 | 3.767 |
| --- | --- | --- | --- |
| BUME_RS19445 | ABC transporter ATP-binding protein | 10.929 | 3.450 |
| BUME_RS11030 | deoxyribose-phosphate aldolase | 10.47 | 3.388 |
| BUME_RS04375 | phage head-tail connector protein | 10.47 | 3.388 |
| BUME_RS19460 | ABC transporter permease subunit | 9.921 | 3.311 |
| BUME_RS19470 | class I SAM-dependent methyltransferase | 9.645 | 3.270 |
| BUME_RS03295 | SDR family oxidoreductase | 9.423 | 3.236 |
| BUME_RS19455 | ABC transporter permease subunit | 8.927 | 3.158 |
| BUME_RS19465 | nickel/metallophore periplasmic binding protein | 8.066 | 3.012 |
| BUME_RS13525 | PqqD family protein | 7.329 | 2.874 |
| BUME_RS15060 | helix-turn-helix transcriptional regulator | 5.46 | 2.449 |
| BUME_RS19295 | MATE efflux family protein | 4.603 | 2.203 |
| BUME_RS05790 | uroporphyrinogen decarboxylase | 4.188 | 2.066 |
| BUME_RS04000 | hypothetical protein | 3.752 | 1.908 |
| BUME_RS19450 | ABC transporter ATP-binding protein | 3.629 | 1.860 |
| BUME_RS18500 | TOBE domain-containing protein | 3.49 | 1.803 |
| BUME_RS19290 | winged helix-turn-helix transcriptional regulator | 3.49 | 1.803 |
| BUME_RS05420 | hypothetical protein | 3.403 | 1.767 |
| BUME_RS15080 | FAD-dependent oxidoreductase | 3.396 | 1.764 |
| BUME_RS15075 | peroxiredoxin | 3.376 | 1.755 |
| BUME_RS11865 | iron ABC transporter permease | 3.35 | 1.744 |
| BUME_RS05875 | ABC transporter substrate-binding protein | 3.193 | 1.675 |
| BUME_RS14805 | demethoxyubiquinone hydroxylase family protein | 3.104 | 1.634 |
| BUME_RS11870 | ABC transporter ATP-binding protein | 3.041 | 1.605 |
| BUME_RS05495 | ketol-acid reductoisomerase | 2.85 | 1.511 |
| BUME_RS11860 | iron ABC transporter substrate-binding protein | 2.745 | 1.457 |
| BUME_RS11885 | ABC transporter substrate-binding protein | 2.692 | 1.429 |
| BUME_RS00995 | hypothetical protein | 2.506 | 1.326 |
| BUME_RS06625 | YlbF family regulator | 2.48 | 1.311 |
| BUME_RS11720 | YbhB/YbcL family Raf kinase inhibitor-like protein | 2.455 | 1.296 |
| BUME_RS14810 | bacteriocin family protein | 2.446 | 1.291 |
| BUME_RS06560 | Rrf2 family transcriptional regulator | 2.317 | 1.212 |
| BUME_RS00540 | YggT family protein | 2.303 | 1.204 |
| BUME_RS10435 | hypothetical protein | 2.303 | 1.204 |
| BUME_RS01025 | hypothetical protein | 2.302 | 1.203 |
| BUME_RS11115 | sulfurtransferase TusA family protein | 2.274 | 1.185 |
| BUME_RS14430 | hypothetical protein | 2.246 | 1.167 |
| BUME_RS10340 | hypothetical protein | 2.243 | 1.166 |
| BUME_RS16655 | cation-translocating P-type ATPase | 2.243 | 1.166 |
| BUME_RS08110 | cold-shock protein | 2.227 | 1.155 |
| BUME_RS00730 | FeoB-associated Cys-rich membrane protein | 2.215 | 1.147 |
| BUME_RS11715 | hypothetical protein | 2.18 | 1.124 |
| BUME_RS10230 | TetR/AcrR family transcriptional regulator | 2.179 | 1.123 |
| BUME_RS16065 | diol dehydratase small subunit | 2.169 | 1.117 |
| BUME_RS11120 | YeeE/YedE family protein | 2.123 | 1.086 |
| BUME_RS18060 | ribonuclease | 2.122 | 1.085 |
| BUME_RS05630 | cation transporter | 2.058 | 1.041 |
| BUME_RS16080 | propanediol utilization microcompartment protein PduB | 2.046 | 1.033 |
| BUME_RS13465 | peptidoglycan DD-metalloendopeptidase family protein | 2.044 | 1.031 |
| BUME_RS00805 | transcriptional repressor | 2.04 | 1.029 |
| BUME_RS16485 | cation-translocating P-type ATPase | 2.01 | 1.007 |
| BUME_RS11050 | hypothetical protein | 2.01 | 1.007 |
| BUME_RS16070 | propanediol/glycerol family dehydratase medium subunit | 2.002 | 1.001 |

Table S3 Significantly down-regulated genes of [*B. methylotrophicum*](http://www.baidu.com/link?url=a08t7ZfLRpoPwIik17uVjRE3V-ht7i5jW_tFgbnBx5kjVx85Ai1twWP8CjUZGbW36uXW93jmoGQM65MFQ_SFjq) in response to lysine addition

| Gene_id | Gene description | Fold Change | Log2 (FC) |
| --- | --- | --- | --- |
| BUME_RS18660 | nucleotide sugar dehydrogenase | 0.031 | -4.998 |
| BUME_RS17665 | acetylornithine transaminase | 0.033 | -4.925 |
| BUME_RS02390 | EamA family transporter | 0.035 | -4.825 |
| BUME_RS17650 | N-acetyl-gamma-glutamyl-phosphate reductase | 0.036 | -4.805 |
| BUME_RS17655 | bifunctional glutamate N-acetyltransferase/amino-acid acetyltransferase ArgJ | 0.039 | -4.667 |
| BUME_RS17660 | acetylglutamate kinase | 0.040 | -4.645 |
| BUME_RS17640 | argininosuccinate lyase | 0.041 | -4.597 |
| BUME_RS08440 | helix-turn-helix transcriptional regulator | 0.044 | -4.519 |
| BUME_RS17645 | argininosuccinate synthase | 0.049 | -4.342 |
| BUME_RS13430 | aminotransferase class IV | 0.052 | -4.256 |
| BUME_RS15990 | hypothetical protein | 0.052 | -4.256 |
| BUME_RS12255 | FAD-binding oxidoreductase | 0.065 | -3.934 |
| BUME_RS02055 | PadR family transcriptional regulator | 0.065 | -3.934 |
| BUME_RS09485 | sodium/proline symporter PutP | 0.069 | -3.848 |
| BUME_RS02700 | pyruvate carboxylase | 0.090 | -3.475 |
| BUME_RS12250 | L-lactate permease | 0.091 | -3.465 |
| BUME_RS09025 | 3-oxoacyl-[acyl-carrier-protein] reductase | 0.109 | -3.203 |
| BUME_RS12260 | electron transfer flavoprotein subunit alpha/FixB family protein | 0.114 | -3.136 |
| BUME_RS18915 | PTS glucitol/sorbitol transporter subunit IIA | 0.121 | -3.049 |
| BUME_RS09030 | ACP S-malonyltransferase | 0.125 | -3.003 |
| BUME_RS00750 | diguanylate cyclase | 0.131 | -2.934 |
| BUME_RS09040 | DUF561 domain-containing protein | 0.134 | -2.902 |
| BUME_RS03350 | DUF2273 domain-containing protein | 0.137 | -2.872 |
| BUME_RS15055 | hypothetical protein | 0.138 | -2.855 |
| BUME_RS06490 | ABC transporter ATP-binding protein | 0.140 | -2.841 |
| BUME_RS18935 | YjbQ family protein | 0.143 | -2.802 |
| BUME_RS12050 | ABC-F family ATP-binding cassette domain-containing protein | 0.144 | -2.799 |
| BUME_RS17835 | 50S ribosomal protein L35 | 0.145 | -2.784 |
| BUME_RS09045 | ketoacyl-ACP synthase III | 0.148 | -2.760 |
| BUME_RS10810 | trimethylamine methyltransferase family protein | 0.150 | -2.741 |
| BUME_RS11570 | N-acetylmannosamine-6-phosphate 2-epimerase | 0.150 | -2.741 |
| BUME_RS09020 | beta-ketoacyl-ACP synthase II | 0.151 | -2.730 |
| BUME_RS09050 | acetyl-CoA carboxylase carboxyltransferase subunit alpha | 0.151 | -2.732 |
| BUME_RS09185 | ABC-2 transporter permease | 0.151 | -2.725 |
| BUME_RS20430 | 30S ribosomal protein S16 | 0.153 | -2.709 |
| BUME_RS03590 | site-specific integrase | 0.154 | -2.699 |
| BUME_RS05535 | MATE family efflux transporter | 0.156 | -2.681 |
| BUME_RS09035 | nitronate monooxygenase | 0.160 | -2.648 |
| BUME_RS04425 | hypothetical protein | 0.165 | -2.597 |
| BUME_RS09055 | acetyl-CoA carboxylase carboxyltransferase subunit beta | 0.168 | -2.575 |
| BUME_RS13180 | transposase | 0.170 | -2.559 |
| BUME_RS15050 | accessory gene regulator B family protein | 0.172 | -2.540 |
| BUME_RS10650 | CRISPR-associated protein Cas4 | 0.174 | -2.519 |
| BUME_RS15395 | transaldolase | 0.174 | -2.519 |
| BUME_RS02045 | DUF4097 family beta strand repeat protein | 0.179 | -2.480 |
| BUME_RS20210 | peptidase M15 | 0.185 | -2.436 |
| BUME_RS13195 | type I restriction endonuclease subunit R | 0.187 | -2.418 |
| BUME_RS09060 | acetyl-CoA carboxylase biotin carboxylase subunit | 0.187 | -2.422 |
| BUME_RS05310 | MerR family transcriptional regulator | 0.188 | -2.410 |
| BUME_RS01005 | site-specific integrase | 0.190 | -2.395 |
| BUME_RS09160 | hypothetical protein | 0.190 | -2.393 |
| BUME_RS01645 | hypothetical protein | 0.191 | -2.385 |
| BUME_RS14820 | hypothetical protein | 0.191 | -2.385 |
| BUME_RS17560 | basic amino acid ABC transporter substrate-binding protein | 0.193 | -2.374 |
| BUME_RS09015 | 3-hydroxyacyl-ACP dehydratase FabZ | 0.193 | -2.374 |
| BUME_RS13190 | restriction endonuclease subunit S | 0.193 | -2.373 |
| BUME_RS11575 | N-acetylneuraminate lyase | 0.194 | -2.367 |
| BUME_RS17830 | 50S ribosomal protein L20 | 0.195 | -2.356 |
| BUME_RS00210 | Coenzyme F420 hydrogenase/dehydrogenase, beta  subunit C-terminal domain | 0.199 | -2.332 |
| BUME_RS13405 | ABC transporter permease | 0.199 | -2.332 |
| BUME_RS02040 | CPBP family intramembrane metalloprotease | 0.203 | -2.303 |
| BUME_RS18920 | PTS glucitol/sorbitol transporter subunit IIB | 0.203 | -2.299 |
| BUME_RS05330 | hypothetical protein | 0.203 | -2.303 |
| BUME_RS20335 | cyclic lactone autoinducer peptide | 0.204 | -2.294 |
| BUME_RS10880 | LysR family transcriptional regulator | 0.205 | -2.286 |
| BUME_RS03585 | RNA-binding transcriptional accessory protein | 0.206 | -2.279 |
| BUME_RS15005 | succinylglutamate desuccinylase/aspartoacylase family protein | 0.206 | -2.281 |
| BUME_RS19925 | GNAT family N-acetyltransferase | 0.206 | -2.282 |
| BUME_RS12410 | DUF86 domain-containing protein | 0.209 | -2.256 |
| BUME_RS06645 | site-specific integrase | 0.209 | -2.256 |
| BUME_RS14210 | arsenical pump-driving ATPase | 0.212 | -2.240 |
| BUME_RS03550 | FAD-dependent oxidoreductase | 0.213 | -2.230 |
| BUME_RS08445 | DUF3169 family protein | 0.220 | -2.182 |
| BUME_RS03390 | cobalamin B12-binding domain-containing protein | 0.220 | -2.182 |
| BUME_RS13400 | ABC transporter ATP-binding protein | 0.224 | -2.156 |
| BUME_RS18080 | pirin family protein | 0.228 | -2.131 |
| BUME_RS15705 | 50S ribosomal protein L17 | 0.233 | -2.099 |
| BUME_RS18940 | triose-phosphate isomerase | 0.233 | -2.100 |
| BUME_RS07960 | hypothetical protein | 0.235 | -2.091 |
| BUME_RS01510 | MarR family transcriptional regulator | 0.236 | -2.081 |
| BUME_RS16515 | molecular chaperone HtpG | 0.238 | -2.070 |
| BUME_RS08035 | ParB N-terminal domain-containing protein | 0.239 | -2.068 |
| BUME_RS03690 | hypothetical protein | 0.240 | -2.058 |
| BUME_RS08280 | type II toxin-antitoxin system RelE/ParE family toxin | 0.240 | -2.059 |
| BUME_RS09070 | acyl carrier protein | 0.241 | -2.054 |
| BUME_RS08390 | Sapep family Mn (^2+^)-dependent dipeptidase | 0.242 | -2.045 |
| BUME_RS02410 | C4-dicarboxylate transporter DcuC | 0.242 | -2.049 |
| BUME_RS09280 | potassium-transporting ATPase subunit KdpB | 0.244 | -2.033 |
| BUME_RS03555 | winged helix-turn-helix transcriptional regulator | 0.245 | -2.030 |
| BUME_RS07945 | site-specific integrase | 0.246 | -2.024 |
| BUME_RS03595 | helix-turn-helix transcriptional regulator | 0.248 | -2.009 |
| BUME_RS16280 | DUF3987 domain-containing protein | 0.249 | -2.008 |
| BUME_RS13245 | aldo/keto reductase | 0.249 | -2.008 |
| BUME_RS15165 | hypothetical protein | 0.251 | -1.993 |
| BUME_RS04670 | putative ABC transporter permease | 0.252 | -1.986 |
| BUME_RS04470 | carboxylating nicotinate-nucleotide diphosphorylase | 0.253 | -1.984 |
| BUME_RS11910 | Lrp/AsnC family transcriptional regulator | 0.253 | -1.982 |
| BUME_RS14215 | metalloregulator ArsR/SmtB family transcription factor | 0.253 | -1.982 |
| BUME_RS08640 | amino acid ABC transporter permease | 0.254 | -1.980 |
| BUME_RS05895 | LytTR family transcriptional regulator | 0.255 | -1.969 |
| BUME_RS12750 | hypothetical protein | 0.255 | -1.973 |
| BUME_RS18325 | FUSC family protein | 0.256 | -1.967 |
| BUME_RS15710 | DNA-directed RNA polymerase subunit alpha | 0.256 | -1.965 |
| BUME_RS08215 | acyltransferase | 0.256 | -1.966 |
| BUME_RS19230 | PASTA domain-containing protein | 0.257 | -1.963 |
| BUME_RS08635 | amino acid ABC transporter ATP-binding protein | 0.257 | -1.962 |
| BUME_RS08155 | LPXTG cell wall anchor domain-containing protein | 0.257 | -1.963 |
| BUME_RS18925 | PTS glucitol/sorbitol transporter subunit IIC | 0.257 | -1.959 |
| BUME_RS16015 | 4Fe-4S dicluster domain-containing protein | 0.258 | -1.954 |
| BUME_RS10545 | sn-glycerol-1-phosphate dehydrogenase | 0.259 | -1.950 |
| BUME_RS19935 | sugar nucleotide-binding protein | 0.262 | -1.934 |
| BUME_RS20770 | hypothetical protein | 0.262 | -1.934 |
| BUME_RS07415 | peptidase domain-containing ABC transporter | 0.262 | -1.934 |
| BUME_RS10000 | LysR family transcriptional regulator | 0.262 | -1.934 |
| BUME_RS15690 | energy-coupling factor transporter transmembrane protein EcfT | 0.264 | -1.924 |
| BUME_RS02640 | 5-formyltetrahydrofolate cyclo-ligase | 0.265 | -1.918 |
| BUME_RS15755 | preprotein translocase subunit SecY | 0.265 | -1.914 |
| BUME_RS10550 | sugar-binding transcriptional regulator | 0.267 | -1.907 |
| BUME_RS12150 | ABC transporter ATP-binding protein | 0.267 | -1.907 |
| BUME_RS08235 | pyridoxal-dependent decarboxylase | 0.268 | -1.901 |
| BUME_RS15760 | 50S ribosomal protein L15 | 0.269 | -1.894 |
| BUME_RS14395 | methionine--tRNA ligase | 0.269 | -1.895 |
| BUME_RS15630 | MarR family transcriptional regulator | 0.270 | -1.888 |
| BUME_RS12265 | electron transfer flavoprotein subunit beta/FixA family protein | 0.271 | -1.885 |
| BUME_RS13185 | SAM-dependent DNA methyltransferase | 0.271 | -1.881 |
| BUME_RS05590 | ABC transporter ATP-binding protein | 0.271 | -1.881 |
| BUME_RS16010 | hypothetical protein | 0.273 | -1.871 |
| BUME_RS03345 | Asp23/Gls24 family envelope stress response protein | 0.274 | -1.868 |
| BUME_RS07605 | tRNA epoxyqueuosine(34) reductase QueG | 0.274 | -1.867 |
| BUME_RS02050 | DUF1700 domain-containing protein | 0.274 | -1.869 |
| BUME_RS12075 | hypothetical protein | 0.276 | -1.859 |
| BUME_RS08890 | ABC transporter ATP-binding protein | 0.277 | -1.854 |
| BUME_RS01795 | GGDEF domain-containing protein | 0.277 | -1.851 |
| BUME_RS01110 | recombinase family protein | 0.278 | -1.845 |
| BUME_RS12830 | dipeptide ABC transporter ATP-binding protein | 0.282 | -1.826 |
| BUME_RS00585 | DNA-directed RNA polymerase subunit omega | 0.282 | -1.827 |
| BUME_RS14160 | hypothetical protein | 0.282 | -1.827 |
| BUME_RS15490 | (4Fe-4S)-binding protein | 0.282 | -1.827 |
| BUME_RS15695 | energy-coupling factor transporter ATPase | 0.283 | -1.823 |
| BUME_RS17005 | sigma-54-dependent Fis family transcriptional regulator | 0.283 | -1.821 |
| BUME_RS16530 | rubrerythrin family protein | 0.284 | -1.816 |
| BUME_RS16770 | Coenzyme F420 hydrogenase/dehydrogenase, beta subunit C-terminal domain | 0.284 | -1.816 |
| BUME_RS11820 | response regulator | 0.286 | -1.806 |
| BUME_RS19910 | DegT/DnrJ/EryC1/StrS family aminotransferase | 0.286 | -1.806 |
| BUME_RS18475 | phosphate ABC transporter substrate-binding protein | 0.286 | -1.806 |
| BUME_RS11195 | chromate transporter | 0.286 | -1.805 |
| BUME_RS14475 | SLC13/DASS family transporter | 0.286 | -1.808 |
| BUME_RS19220 | phospho-N-acetylmuramoyl-pentapeptide-transferase | 0.287 | -1.800 |
| BUME_RS05565 | phosphate signaling complex protein PhoU | 0.287 | -1.801 |
| BUME_RS08030 | DUF3440 domain-containing protein | 0.288 | -1.794 |
| BUME_RS08150 | isopeptide-forming domain-containing fimbrial protein | 0.288 | -1.796 |
| BUME_RS20140 | WYL domain-containing protein | 0.289 | -1.792 |
| BUME_RS18650 | class I SAM-dependent methyltransferase | 0.289 | -1.792 |
| BUME_RS15715 | 30S ribosomal protein S4 | 0.290 | -1.788 |
| BUME_RS15725 | 30S ribosomal protein S13 | 0.291 | -1.782 |
| BUME_RS11190 | chromate transporter | 0.291 | -1.782 |
| BUME_RS08145 | class C sortase | 0.291 | -1.782 |
| BUME_RS15720 | 30S ribosomal protein S11 | 0.292 | -1.774 |
| BUME_RS10785 | hypothetical protein | 0.293 | -1.770 |
| BUME_RS04845 | 30S ribosomal protein S16 | 0.294 | -1.768 |
| BUME_RS05225 | AAA family ATPase | 0.294 | -1.765 |
| BUME_RS12980 | hypothetical protein | 0.294 | -1.764 |
| BUME_RS00315 | aldehyde ferredoxin oxidoreductase | 0.294 | -1.764 |
| BUME_RS07595 | endopeptidase La | 0.295 | -1.761 |
| BUME_RS08290 | viroplasmin family protein | 0.296 | -1.759 |
| BUME_RS19225 | UDP-N-acetylmuramoyl-tripeptide--D-alanyl-D-alanine ligase | 0.297 | -1.753 |
| BUME_RS09005 | response regulator transcription factor | 0.297 | -1.750 |
| BUME_RS10705 | cysteine synthase A | 0.297 | -1.750 |
| BUME_RS08020 | choloylglycine hydrolase family protein | 0.298 | -1.748 |
| BUME_RS13800 | hypothetical protein | 0.298 | -1.744 |
| BUME_RS15700 | energy-coupling factor transporter ATPase | 0.299 | -1.742 |
| BUME_RS18855 | dihydropyrimidinase | 0.300 | -1.738 |
| BUME_RS19950 | hypothetical protein | 0.301 | -1.733 |
| BUME_RS14265 | hypothetical protein | 0.302 | -1.730 |
| BUME_RS15770 | 30S ribosomal protein S5 | 0.302 | -1.725 |
| BUME_RS08580 | LysR family transcriptional regulator | 0.302 | -1.727 |
| BUME_RS08025 | YSIRK-targeted surface antigen transcriptional regulator | 0.303 | -1.723 |
| BUME_RS20555 | hypothetical protein | 0.304 | -1.718 |
| BUME_RS06035 | YihY/virulence factor BrkB family protein | 0.304 | -1.716 |
| BUME_RS12420 | type IA DNA topoisomerase | 0.304 | -1.718 |
| BUME_RS08645 | amino acid ABC transporter substrate-binding protein | 0.305 | -1.713 |
| BUME_RS18930 | transcriptional regulator GutM | 0.305 | -1.711 |
| BUME_RS10775 | anion permease | 0.305 | -1.711 |
| BUME_RS19945 | acyltransferase | 0.305 | -1.715 |
| BUME_RS11165 | FAD-dependent oxidoreductase | 0.306 | -1.710 |
| BUME_RS16160 | Cna B-type domain-containing protein | 0.307 | -1.702 |
| BUME_RS11180 | ABC transporter substrate-binding protein | 0.307 | -1.702 |
| BUME_RS06145 | hypothetical protein | 0.308 | -1.698 |
| BUME_RS15750 | adenylate kinase | 0.308 | -1.698 |
| BUME_RS15530 | hypothetical protein | 0.308 | -1.701 |
| BUME_RS19965 | acyltransferase | 0.308 | -1.699 |
| BUME_RS04310 | AP2 domain-containing protein | 0.309 | -1.695 |
| BUME_RS00410 | 2-amino-4-hydroxy-6-hydroxymethyldihydropteridine diphosphokinase | 0.310 | -1.691 |
| BUME_RS11815 | radical SAM protein | 0.310 | -1.690 |
| BUME_RS05815 | hypothetical protein | 0.310 | -1.689 |
| BUME_RS07490 | heat-inducible transcription repressor HrcA | 0.312 | -1.678 |
| BUME_RS16455 | elongation factor G | 0.313 | -1.676 |
| BUME_RS03505 | collagen binding domain-containing protein | 0.313 | -1.675 |
| BUME_RS13635 | sensor histidine kinase | 0.314 | -1.672 |
| BUME_RS12660 | recombinase family protein | 0.314 | -1.672 |
| BUME_RS19955 | hypothetical protein | 0.314 | -1.671 |
| BUME_RS13260 | potassium-transporting ATPase subunit KdpB | 0.314 | -1.671 |
| BUME_RS13020 | site-specific integrase | 0.314 | -1.671 |
| BUME_RS12000 | GntR family transcriptional regulator | 0.315 | -1.668 |
| BUME_RS01580 | glutamate:gamma-aminobutyrate antiporter | 0.316 | -1.661 |
| BUME_RS18945 | class II fructose-bisphosphate aldolase | 0.316 | -1.661 |
| BUME_RS00220 | hypothetical protein | 0.317 | -1.658 |
| BUME_RS03850 | efflux RND transporter periplasmic adaptor subunit | 0.317 | -1.656 |
| BUME_RS07315 | 50S ribosomal protein L1 | 0.318 | -1.652 |
| BUME_RS07600 | YihA family ribosome biogenesis GTP-binding protein | 0.318 | -1.654 |
| BUME_RS15730 | 50S ribosomal protein L36 | 0.319 | -1.650 |
| BUME_RS15345 | GntR family transcriptional regulator | 0.319 | -1.648 |
| BUME_RS00695 | DMT family transporter | 0.319 | -1.649 |
| BUME_RS06100 | EpsG family protein | 0.320 | -1.642 |
| BUME_RS19215 | UDP-N-acetylmuramoyl-L-alanine--D-glutamate ligase | 0.320 | -1.643 |
| BUME_RS13085 | SDR family oxidoreductase | 0.320 | -1.642 |
| BUME_RS08335 | anaerobic sulfite reductase subunit B | 0.320 | -1.644 |
| BUME_RS19680 | LexA family transcriptional regulator | 0.322 | -1.636 |
| BUME_RS01010 | DNA adenine methylase | 0.323 | -1.629 |
| BUME_RS11970 | response regulator transcription factor | 0.323 | -1.630 |
| BUME_RS12665 | YSIRK-type signal peptide-containing protein | 0.324 | -1.628 |
| BUME_RS19050 | formate C-acetyltransferase/glycerol dehydratase family glycyl radical enzyme | 0.324 | -1.625 |
| BUME_RS02915 | serine hydrolase | 0.324 | -1.626 |
| BUME_RS07115 | nickel-dependent lactate racemase | 0.325 | -1.621 |
| BUME_RS15685 | tRNA pseudouridine(38-40) synthase TruA | 0.325 | -1.621 |
| BUME_RS04440 | SPP1 phage holin family protein | 0.325 | -1.622 |
| BUME_RS03360 | hypothetical protein | 0.326 | -1.616 |
| BUME_RS04950 | DUF2089 domain-containing protein | 0.326 | -1.617 |
| BUME_RS19365 | UDP-N-acetylglucosamine 2-epimerase (non-hydrolyzing) | 0.326 | -1.617 |
| BUME_RS20890 | hypothetical protein | 0.326 | -1.618 |
| BUME_RS18445 | oleate hydratase | 0.327 | -1.613 |
| BUME_RS11670 | xylulose kinase | 0.329 | -1.605 |
| BUME_RS18775 | sulfurtransferase-like selenium metabolism protein YedF | 0.329 | -1.605 |
| BUME_RS07335 | hypothetical protein | 0.330 | -1.598 |
| BUME_RS00555 | hypothetical protein | 0.330 | -1.600 |
| BUME_RS01225 | DNA repair protein RecN | 0.331 | -1.596 |
| BUME_RS09565 | hypothetical protein | 0.331 | -1.597 |
| BUME_RS14730 | hypothetical protein | 0.332 | -1.593 |
| BUME_RS06250 | 1-acyl-sn-glycerol-3-phosphate acyltransferase | 0.332 | -1.593 |
| BUME_RS18680 | UDP-glucose 4-epimerase GalE | 0.332 | -1.591 |
| BUME_RS08160 | ppGpp-regulated growth inhibitor | 0.333 | -1.586 |
| BUME_RS00215 | polysaccharide pyruvyl transferase family protein | 0.334 | -1.584 |
| BUME_RS16315 | hypothetical protein | 0.334 | -1.582 |
| BUME_RS08285 | hypothetical protein | 0.334 | -1.580 |
| BUME_RS17075 | tRNA (adenine(22)-N(1))-methyltransferase TrmK | 0.335 | -1.580 |
| BUME_RS10850 | surface proteins containing Ig-like domains | 0.335 | -1.576 |
| BUME_RS16475 | DNA-directed RNA polymerase subunit beta' | 0.336 | -1.572 |
| BUME_RS04825 | chromosome segregation protein SMC | 0.336 | -1.574 |
| BUME_RS15790 | type Z 30S ribosomal protein S14 | 0.336 | -1.572 |
| BUME_RS02665 | dicarboxylate/amino acid:cation symporter | 0.336 | -1.572 |
| BUME_RS18820 | carbamate kinase | 0.336 | -1.573 |
| BUME_RS19210 | putative lipid II flippase FtsW | 0.337 | -1.570 |
| BUME_RS20765 | hypothetical protein | 0.337 | -1.569 |
| BUME_RS15735 | translation initiation factor IF-1 | 0.337 | -1.567 |
| BUME_RS16705 | YdcF family protein | 0.337 | -1.570 |
| BUME_RS09520 | hypothetical protein | 0.337 | -1.569 |
| BUME_RS00190 | hypothetical protein | 0.337 | -1.571 |
| BUME_RS00725 | flavodoxin | 0.338 | -1.567 |
| BUME_RS10700 | O-acetylhomoserine aminocarboxypropyltransferase/cysteine synthase | 0.338 | -1.566 |
| BUME_RS18355 | SWIM zinc finger protein | 0.338 | -1.564 |
| BUME_RS14610 | ABC-2 transporter permease | 0.338 | -1.566 |
| BUME_RS17405 | DMT family transporter | 0.338 | -1.566 |
| BUME_RS13650 | chaperonin GroEL | 0.339 | -1.562 |
| BUME_RS09995 | hypothetical protein | 0.339 | -1.561 |
| BUME_RS11185 | hypothetical protein | 0.339 | -1.559 |
| BUME_RS18790 | EF2563 family selenium-dependent molybdenum hydroxylase system protein | 0.339 | -1.562 |
| BUME_RS07330 | proline--tRNA ligase | 0.340 | -1.557 |
| BUME_RS11330 | 30S ribosomal protein S18 | 0.340 | -1.558 |
| BUME_RS08385 | oligopeptide transporter, OPT family | 0.340 | -1.555 |
| BUME_RS20020 | BCCT family transporter | 0.340 | -1.558 |
| BUME_RS03610 | linear amide C-N hydrolase | 0.341 | -1.552 |
| BUME_RS19250 | ACT domain-containing protein | 0.341 | -1.550 |
| BUME_RS04275 | hypothetical protein | 0.341 | -1.553 |
| BUME_RS09140 | glycerol-3-phosphate responsive antiterminator | 0.341 | -1.553 |
| BUME_RS19235 | hypothetical protein | 0.343 | -1.545 |
| BUME_RS02395 | 2-oxoacid: acceptor oxidoreductase subunit alpha | 0.343 | -1.543 |
| BUME_RS15065 | LysR family transcriptional regulator | 0.343 | -1.545 |
| BUME_RS16805 | N-acetyltransferase | 0.344 | -1.538 |
| BUME_RS16465 | 30S ribosomal protein S12 | 0.344 | -1.540 |
| BUME_RS01515 | DegV family protein | 0.344 | -1.541 |
| BUME_RS11810 | TetR/AcrR family transcriptional regulator | 0.344 | -1.541 |
| BUME_RS00490 | MATE family efflux transporter | 0.345 | -1.534 |
| BUME_RS18800 | putative selenium-dependent hydroxylase accessory protein YqeC | 0.345 | -1.534 |
| BUME_RS19800 | DNA mismatch repair protein MutS | 0.346 | -1.533 |
| BUME_RS10540 | HAD-IIA family hydrolase | 0.346 | -1.529 |
| BUME_RS07510 | dihydroxyacetone kinase transcriptional activator DhaS | 0.346 | -1.533 |
| BUME_RS18910 | hypothetical protein | 0.346 | -1.532 |
| BUME_RS15745 | type I methionyl aminopeptidase | 0.347 | -1.526 |
| BUME_RS01870 | 30S ribosomal protein S21 | 0.349 | -1.519 |
| BUME_RS04965 | hypothetical protein | 0.349 | -1.519 |
| BUME_RS18195 | DUF4147 domain-containing protein | 0.349 | -1.519 |
| BUME_RS19360 | Gfo/Idh/MocA family oxidoreductase | 0.349 | -1.519 |
| BUME_RS07640 | D-tyrosyl-tRNA (Tyr) deacylase | 0.349 | -1.519 |
| BUME_RS12365 | hypothetical protein | 0.349 | -1.519 |
| BUME_RS11980 | ABC transporter permease | 0.349 | -1.519 |
| BUME_RS19940 | UDP-N-acetylglucosamine 4,6-dehydratase(inverting) | 0.349 | -1.519 |
| BUME_RS00510 | 2-hydroxyacid dehydrogenase | 0.350 | -1.516 |
| BUME_RS18655 | SDR family oxidoreductase | 0.350 | -1.513 |
| BUME_RS07590 | ATP-dependent Clp protease ATP-binding subunit ClpX | 0.351 | -1.509 |
| BUME_RS04465 | L-aspartate oxidase | 0.353 | -1.501 |
| BUME_RS12305 | xylulokinase | 0.353 | -1.503 |
| BUME_RS05545 | phosphate ABC transporter substrate-binding protein | 0.354 | -1.499 |
| BUME_RS10010 | hypothetical protein | 0.354 | -1.498 |
| BUME_RS12315 | GntR family transcriptional regulator | 0.355 | -1.495 |
| BUME_RS00950 | PTS transporter subunit IIABC | 0.355 | -1.492 |
| BUME_RS02695 | CBS domain-containing protein | 0.355 | -1.495 |
| BUME_RS09360 | hypothetical protein | 0.355 | -1.496 |
| BUME_RS07135 | DUF2776 domain-containing protein | 0.356 | -1.489 |
| BUME_RS01015 | AAA family ATPase | 0.356 | -1.490 |
| BUME_RS04475 | transcription repressor NadR | 0.356 | -1.492 |
| BUME_RS19980 | glycosyltransferase family 4 protein | 0.356 | -1.492 |
| BUME_RS17015 | cyclase family protein | 0.356 | -1.490 |
| BUME_RS17435 | sigma-70 family RNA polymerase sigma factor | 0.356 | -1.488 |
| BUME_RS06005 | glycoside hydrolase family 13 protein | 0.358 | -1.481 |
| BUME_RS15175 | lysine--tRNA ligase | 0.358 | -1.481 |
| BUME_RS16460 | 30S ribosomal protein S7 | 0.358 | -1.481 |
| BUME_RS06345 | transcription-repair coupling factor | 0.358 | -1.480 |
| BUME_RS09200 | hydroxyacid dehydrogenase | 0.358 | -1.481 |
| BUME_RS08040 | DeoR/GlpR transcriptional regulator | 0.359 | -1.477 |
| BUME_RS15775 | 50S ribosomal protein L18 | 0.359 | -1.477 |
| BUME_RS17385 | TIGR04076 family protein | 0.359 | -1.480 |
| BUME_RS10755 | hypothetical protein | 0.359 | -1.478 |
| BUME_RS15465 | KUP/HAK/KT family potassium transporter | 0.360 | -1.475 |
| BUME_RS15780 | 50S ribosomal protein L6 | 0.361 | -1.468 |
| BUME_RS19395 | glycosyltransferase | 0.361 | -1.469 |
| BUME_RS19930 | N-acetylneuraminate synthase family protein | 0.361 | -1.469 |
| BUME_RS02035 | radical SAM protein | 0.361 | -1.469 |
| BUME_RS07310 | 50S ribosomal protein L11 | 0.362 | -1.464 |
| BUME_RS09065 | acetyl-CoA carboxylase biotin carboxyl carrier protein | 0.362 | -1.465 |
| BUME_RS06245 | glycosyltransferase | 0.362 | -1.465 |
| BUME_RS06460 | CoA-transferase | 0.362 | -1.466 |
| BUME_RS05945 | radical SAM protein | 0.362 | -1.466 |
| BUME_RS18785 | aminotransferase class V-fold PLP-dependent enzyme | 0.362 | -1.466 |
| BUME_RS05320 | repressor LexA | 0.362 | -1.467 |
| BUME_RS13655 | co-chaperone GroES | 0.363 | -1.464 |
| BUME_RS20360 | glycyl-radical enzyme activating protein | 0.363 | -1.461 |
| BUME_RS16785 | WxcM-like domain-containing protein | 0.363 | -1.462 |
| BUME_RS14595 | MATE family efflux transporter | 0.363 | -1.461 |
| BUME_RS11325 | single-stranded DNA-binding protein | 0.364 | -1.459 |
| BUME_RS07350 | DUF2284 domain-containing protein | 0.364 | -1.459 |
| BUME_RS02575 | sugar phosphate isomerase/epimerase | 0.365 | -1.454 |
| BUME_RS16005 | BMC domain-containing protein | 0.366 | -1.451 |
| BUME_RS16090 | response regulator | 0.366 | -1.449 |
| BUME_RS14540 | 4Fe-4S binding protein | 0.366 | -1.448 |
| BUME_RS16795 | acyltransferase | 0.367 | -1.447 |
| BUME_RS08325 | MFS transporter | 0.368 | -1.443 |
| BUME_RS15470 | class I SAM-dependent methyltransferase | 0.368 | -1.442 |
| BUME_RS18760 | XdhC family protein | 0.368 | -1.442 |
| BUME_RS14590 | MarR family transcriptional regulator | 0.368 | -1.441 |
| BUME_RS05575 | hypothetical protein | 0.369 | -1.440 |
| BUME_RS01290 | GNAT family N-acetyltransferase | 0.369 | -1.440 |
| BUME_RS01235 | NAD (+)/NADH kinase | 0.370 | -1.433 |
| BUME_RS12145 | putative ABC exporter domain-containing protein | 0.370 | -1.434 |
| BUME_RS15035 | MATE family efflux transporter | 0.370 | -1.433 |
| BUME_RS03355 | alkaline shock response membrane anchor protein AmaP | 0.371 | -1.430 |
| BUME_RS10710 | homoserine O-succinyltransferase | 0.371 | -1.429 |
| BUME_RS18605 | NAD(P)-dependent oxidoreductase | 0.371 | -1.431 |
| BUME_RS18850 | putative selenate reductase subunit YgfK | 0.372 | -1.428 |
| BUME_RS11320 | 30S ribosomal protein S6 | 0.372 | -1.426 |
| BUME_RS02660 | purine permease | 0.372 | -1.427 |
| BUME_RS04955 | hypothetical protein | 0.372 | -1.425 |
| BUME_RS05200 | 2,3-diphosphoglycerate-dependent phosphoglycerate mutase | 0.373 | -1.424 |
| BUME_RS03165 | GGDEF domain-containing protein | 0.373 | -1.424 |
| BUME_RS09735 | helix-turn-helix transcriptional regulator | 0.373 | -1.424 |
| BUME_RS01355 | DUF2268 domain-containing protein | 0.373 | -1.423 |
| BUME_RS12060 | GIY-YIG nuclease family protein | 0.373 | -1.423 |
| BUME_RS15195 | adenylosuccinate synthase | 0.374 | -1.418 |
| BUME_RS13155 | EAL domain-containing protein | 0.374 | -1.421 |
| BUME_RS10765 | anion permease | 0.374 | -1.419 |
| BUME_RS03975 | PTS mannitol transporter subunit IICBA | 0.375 | -1.417 |
| BUME_RS02705 | chorismate mutase | 0.375 | -1.417 |
| BUME_RS04120 | AraC family transcriptional regulator | 0.375 | -1.414 |
| BUME_RS07545 | cupin domain-containing protein | 0.376 | -1.413 |
| BUME_RS14515 | L-serine ammonia-lyase, iron-sulfur-dependent, subunit alpha | 0.376 | -1.411 |
| BUME_RS10305 | hypothetical protein | 0.376 | -1.411 |
| BUME_RS08625 | helix-hairpin-helix domain-containing protein | 0.376 | -1.410 |
| BUME_RS09595 | hypothetical protein | 0.376 | -1.412 |
| BUME_RS18685 | hypothetical protein | 0.376 | -1.412 |
| BUME_RS07530 | DNA repair protein RadC | 0.377 | -1.406 |
| BUME_RS16800 | DegT/DnrJ/EryC1/StrS family aminotransferase | 0.377 | -1.406 |
| BUME_RS05555 | phosphate ABC transporter permease PstA | 0.377 | -1.407 |
| BUME_RS13660 | type 1 glutamine amidotransferase domain-containing protein | 0.377 | -1.407 |
| BUME_RS06255 | rubredoxin oxidoreductase (desulfoferrodoxin) | 0.378 | -1.403 |
| BUME_RS15190 | glutamine-hydrolyzing carbamoyl-phosphate synthase small subunit | 0.378 | -1.404 |
| BUME_RS14735 | PadR family transcriptional regulator | 0.379 | -1.401 |
| BUME_RS18825 | knotted carbamoyltransferase YgeW | 0.379 | -1.401 |
| BUME_RS20225 | membrane protein insertion efficiency factor YidD | 0.379 | -1.401 |
| BUME_RS19055 | glycerol kinase GlpK | 0.380 | -1.394 |
| BUME_RS01865 | GatB/YqeY domain-containing protein | 0.380 | -1.396 |
| BUME_RS12430 | helix-turn-helix transcriptional regulator | 0.380 | -1.394 |
| BUME_RS07540 | PBP1A family penicillin-binding protein | 0.381 | -1.392 |
| BUME_RS10235 | hypothetical protein | 0.381 | -1.393 |
| BUME_RS10695 | HD domain-containing protein | 0.381 | -1.391 |
| BUME_RS05070 | GntR family transcriptional regulator | 0.381 | -1.394 |
| BUME_RS01230 | arginine repressor | 0.381 | -1.393 |
| BUME_RS02920 | sodium-translocating pyrophosphatase | 0.382 | -1.387 |
| BUME_RS10535 | hypothetical protein | 0.382 | -1.389 |
| BUME_RS17710 | V-type ATP synthase subunit D | 0.382 | -1.389 |
| BUME_RS17040 | TetR/AcrR family transcriptional regulator | 0.383 | -1.384 |
| BUME_RS19580 | DUF2975 domain-containing protein | 0.383 | -1.384 |
| BUME_RS02925 | preprotein translocase subunit SecG | 0.384 | -1.382 |
| BUME_RS12055 | hypothetical protein | 0.384 | -1.382 |
| BUME_RS15660 | DeoR/GlpR transcriptional regulator | 0.384 | -1.383 |
| BUME_RS06090 | glycosyltransferase family 2 protein | 0.385 | -1.376 |
| BUME_RS18745 | molybdate ABC transporter substrate-binding protein | 0.385 | -1.377 |
| BUME_RS17070 | RNA polymerase sigma factor RpoD | 0.386 | -1.374 |
| BUME_RS10520 | PTS glucitol/sorbitol transporter subunit IIA | 0.386 | -1.372 |
| BUME_RS02505 | GntR family transcriptional regulator | 0.387 | -1.371 |
| BUME_RS16775 | glycosyltransferase | 0.387 | -1.368 |
| BUME_RS07825 | FAD-dependent oxidoreductase | 0.388 | -1.365 |
| BUME_RS19245 | division/cell wall cluster transcriptional repressor MraZ | 0.388 | -1.364 |
| BUME_RS04820 | ribonuclease III | 0.388 | -1.365 |
| BUME_RS06230 | DUF488 family protein | 0.388 | -1.367 |
| BUME_RS20375 | oligosaccharide flippase family protein | 0.388 | -1.365 |
| BUME_RS10670 | hypothetical protein | 0.389 | -1.362 |
| BUME_RS05335 | ImmA/IrrE family metallo-endopeptidase | 0.389 | -1.364 |
| BUME_RS13645 | response regulator transcription factor | 0.389 | -1.362 |
| BUME_RS04810 | phosphate acyltransferase PlsX | 0.390 | -1.359 |
| BUME_RS10845 | Ig-like domain-containing protein | 0.390 | -1.357 |
| BUME_RS01310 | PhzF family phenazine biosynthesis protein | 0.390 | -1.358 |
| BUME_RS12045 | Cof-type HAD-IIB family hydrolase | 0.390 | -1.357 |
| BUME_RS14370 | GntR family transcriptional regulator | 0.390 | -1.357 |
| BUME_RS11540 | exonuclease SbcCD subunit D | 0.391 | -1.356 |
| BUME_RS20200 | (deoxy)nucleoside triphosphate pyrophosphohydrolase | 0.391 | -1.356 |
| BUME_RS19915 | glycosyltransferase family protein | 0.391 | -1.353 |
| BUME_RS15680 | 50S ribosomal protein L13 | 0.392 | -1.352 |
| BUME_RS11545 | AAA family ATPase | 0.392 | -1.349 |
| BUME_RS01180 | LacI family DNA-binding transcriptional regulator | 0.392 | -1.352 |
| BUME_RS02400 | 2-oxoacid: ferredoxin oxidoreductase subunit beta | 0.393 | -1.348 |
| BUME_RS10095 | YdcF family protein | 0.393 | -1.348 |
| BUME_RS11255 | ribonuclease P protein component | 0.393 | -1.346 |
| BUME_RS15545 | DUF3987 domain-containing protein | 0.393 | -1.347 |
| BUME_RS08190 | oligosaccharide flippase family protein | 0.393 | -1.349 |
| BUME_RS07580 | trigger factor | 0.394 | -1.345 |
| BUME_RS12120 | ABC transporter ATP-binding protein | 0.394 | -1.344 |
| BUME_RS17675 | V-type ATP synthase subunit I | 0.395 | -1.340 |
| BUME_RS15835 | 30S ribosomal protein S19 | 0.395 | -1.340 |
| BUME_RS11260 | membrane protein insertase YidC | 0.396 | -1.337 |
| BUME_RS00325 | sodium-dependent transporter | 0.396 | -1.337 |
| BUME_RS00275 | ATP-binding protein | 0.396 | -1.337 |
| BUME_RS06040 | YihY/virulence factor BrkB family protein | 0.397 | -1.332 |
| BUME_RS04860 | tRNA (guanosine(37)-N1)-methyltransferase TrmD | 0.397 | -1.334 |
| BUME_RS15670 | xylulokinase | 0.397 | -1.332 |
| BUME_RS07980 | M18 family aminopeptidase | 0.398 | -1.329 |
| BUME_RS06830 | trimethylamine methyltransferase family protein | 0.398 | -1.330 |
| BUME_RS05015 | diacylglycerol kinase family protein | 0.398 | -1.329 |
| BUME_RS13600 | DUF1538 domain-containing protein | 0.399 | -1.326 |
| BUME_RS04690 | ribonuclease HI | 0.399 | -1.327 |
| BUME_RS02965 | shikimate kinase | 0.399 | -1.325 |
| BUME_RS12835 | ABC transporter ATP-binding protein | 0.400 | -1.322 |
| BUME_RS15185 | carbamoyl-phosphate synthase large subunit | 0.400 | -1.323 |
| BUME_RS19390 | O-antigen ligase family protein | 0.400 | -1.322 |
| BUME_RS15460 | hypothetical protein | 0.400 | -1.321 |
| BUME_RS16780 | oligosaccharide flippase family protein | 0.401 | -1.317 |
| BUME_RS08765 | sodium:alanine symporter family protein | 0.401 | -1.318 |
| BUME_RS16645 | HAD family hydrolase | 0.401 | -1.318 |
| BUME_RS06045 | aconitate hydratase | 0.402 | -1.315 |
| BUME_RS00225 | glycosyltransferase | 0.402 | -1.314 |
| BUME_RS03675 | Rrf2 family transcriptional regulator | 0.403 | -1.312 |
| BUME_RS13095 | 5'-nucleotidase C-terminal domain-containing protein | 0.403 | -1.310 |
| BUME_RS17870 | DUF3795 domain-containing protein | 0.403 | -1.310 |
| BUME_RS05300 | hypothetical protein | 0.403 | -1.310 |
| BUME_RS08210 | hypothetical protein | 0.403 | -1.312 |
| BUME_RS05105 | LysR family transcriptional regulator | 0.403 | -1.312 |
| BUME_RS17840 | translation initiation factor IF-3 | 0.404 | -1.309 |
| BUME_RS08715 | redox-regulated ATPase YchF | 0.404 | -1.308 |
| BUME_RS04830 | signal recognition particle-docking protein FtsY | 0.404 | -1.309 |
| BUME_RS18795 | nucleotidyltransferase family protein | 0.404 | -1.307 |
| BUME_RS04225 | 3'-5' exoribonuclease | 0.405 | -1.305 |
| BUME_RS19240 | 16S rRNA (cytosine(1402)-N(4))-methyltransferase RsmH | 0.406 | -1.300 |
| BUME_RS01875 | glutathione peroxidase | 0.406 | -1.299 |
| BUME_RS05560 | phosphate ABC transporter ATP-binding protein | 0.406 | -1.301 |
| BUME_RS03535 | sugar-binding transcriptional regulator | 0.406 | -1.301 |
| BUME_RS10790 | hypothetical protein | 0.406 | -1.299 |
| BUME_RS09000 | HAMP domain-containing histidine kinase | 0.408 | -1.295 |
| BUME_RS12435 | hypothetical protein | 0.408 | -1.295 |
| BUME_RS12935 | bifunctional phosphoribosylaminoimidazolecarboxamide formyltransferase/IMP cyclohydrolase | 0.409 | -1.290 |
| BUME_RS16590 | DMT family transporter | 0.409 | -1.288 |
| BUME_RS09290 | hypothetical protein | 0.410 | -1.286 |
| BUME_RS17400 | CGNR zinc finger domain-containing protein | 0.410 | -1.287 |
| BUME_RS06340 | aminoacyl-tRNA hydrolase | 0.410 | -1.286 |
| BUME_RS07355 | hypothetical protein | 0.411 | -1.282 |
| BUME_RS14545 | LysR family transcriptional regulator | 0.411 | -1.284 |
| BUME_RS10955 | sigma 54-interacting transcriptional regulator | 0.412 | -1.278 |
| BUME_RS15315 | energy-coupling factor ABC transporter permease | 0.412 | -1.280 |
| BUME_RS10005 | hypothetical protein | 0.413 | -1.275 |
| BUME_RS08365 | response regulator transcription factor | 0.413 | -1.274 |
| BUME_RS10820 | cobalamin B12-binding domain-containing protein | 0.413 | -1.276 |
| BUME_RS06010 | glycogen synthase GlgA | 0.414 | -1.272 |
| BUME_RS17080 | Nif3-like dinuclear metal center hexameric protein | 0.414 | -1.271 |
| BUME_RS02210 | hypothetical protein | 0.414 | -1.273 |
| BUME_RS14655 | MerR family transcriptional regulator | 0.414 | -1.271 |
| BUME_RS03770 | IS3 family transposase | 0.414 | -1.271 |
| BUME_RS17615 | hypothetical protein | 0.415 | -1.269 |
| BUME_RS20330 | acyl-CoA dehydrogenase family protein | 0.416 | -1.266 |
| BUME_RS02685 | hypothetical protein | 0.416 | -1.267 |
| BUME_RS08345 | SdpI family protein | 0.416 | -1.267 |
| BUME_RS14615 | DUF3795 domain-containing protein | 0.416 | -1.264 |
| BUME_RS02300 | elongation factor G | 0.417 | -1.263 |
| BUME_RS07740 | 30S ribosomal protein S20 | 0.417 | -1.261 |
| BUME_RS06310 | hypothetical protein | 0.417 | -1.263 |
| BUME_RS19060 | DeoR/GlpR transcriptional regulator | 0.418 | -1.259 |
| BUME_RS17270 | YhgE/Pip domain-containing protein | 0.419 | -1.256 |
| BUME_RS04665 | ferrous iron transport protein B | 0.419 | -1.257 |
| BUME_RS06465 | acyl-CoA dehydrogenase family protein | 0.419 | -1.256 |
| BUME_RS18615 | CDP-glucose 4,6-dehydratase | 0.419 | -1.256 |
| BUME_RS05480 | ABC transporter permease subunit | 0.419 | -1.256 |
| BUME_RS13150 | hypothetical protein | 0.419 | -1.256 |
| BUME_RS05235 | relaxase/mobilization nuclease domain-containing protein | 0.419 | -1.256 |
| BUME_RS07280 | hypothetical protein | 0.419 | -1.256 |
| BUME_RS04890 | YraN family protein | 0.419 | -1.256 |
| BUME_RS15920 | histidine--tRNA ligase | 0.420 | -1.253 |
| BUME_RS17855 | FUSC family protein | 0.420 | -1.251 |
| BUME_RS05475 | ABC transporter substrate-binding protein | 0.420 | -1.250 |
| BUME_RS09860 | carbohydrate kinase | 0.420 | -1.251 |
| BUME_RS16450 | elongation factor Tu | 0.421 | -1.249 |
| BUME_RS15740 | KOW domain-containing RNA-binding protein | 0.421 | -1.247 |
| BUME_RS00920 | aspartate kinase | 0.421 | -1.248 |
| BUME_RS19160 | site-2 protease family protein | 0.421 | -1.248 |
| BUME_RS15785 | 30S ribosomal protein S8 | 0.422 | -1.245 |
| BUME_RS04840 | signal recognition particle protein | 0.422 | -1.244 |
| BUME_RS11100 | LysR family transcriptional regulator | 0.422 | -1.244 |
| BUME_RS07275 | NAD(P)-dependent oxidoreductase | 0.422 | -1.246 |
| BUME_RS14185 | NUDIX hydrolase | 0.422 | -1.246 |
| BUME_RS03620 | TetR/AcrR family transcriptional regulator | 0.423 | -1.242 |
| BUME_RS20010 | APC family permease | 0.423 | -1.242 |
| BUME_RS17240 | ABC transporter ATP-binding protein | 0.423 | -1.242 |
| BUME_RS09010 | valine--tRNA ligase | 0.424 | -1.237 |
| BUME_RS06290 | hypothetical protein | 0.424 | -1.237 |
| BUME_RS05610 | redoxin domain-containing protein | 0.424 | -1.238 |
| BUME_RS02490 | bifunctional precorrin-2 dehydrogenase/sirohydrochlorin ferrochelatase | 0.424 | -1.239 |
| BUME_RS15610 | diguanylate cyclase | 0.425 | -1.234 |
| BUME_RS06575 | AI-2E family transporter | 0.425 | -1.235 |
| BUME_RS11785 | copper-translocating P-type ATPase | 0.425 | -1.235 |
| BUME_RS19920 | aldo/keto reductase | 0.425 | -1.233 |
| BUME_RS07505 | molecular chaperone DnaJ | 0.426 | -1.230 |
| BUME_RS08630 | histidinol-phosphatase HisJ | 0.426 | -1.231 |
| BUME_RS01130 | AraC family transcriptional regulator | 0.426 | -1.230 |
| BUME_RS10525 | PTS glucitol/sorbitol transporter subunit IIB | 0.426 | -1.230 |
| BUME_RS11550 | flavodoxin family protein | 0.427 | -1.229 |
| BUME_RS14625 | sigma 54-interacting transcriptional regulator | 0.427 | -1.228 |
| BUME_RS06960 | thioredoxin family protein | 0.427 | -1.227 |
| BUME_RS02060 | GntR family transcriptional regulator | 0.427 | -1.227 |
| BUME_RS19370 | nucleotide sugar dehydrogenase | 0.427 | -1.229 |
| BUME_RS07495 | nucleotide exchange factor GrpE | 0.428 | -1.225 |
| BUME_RS05620 | ZIP family metal transporter | 0.428 | -1.226 |
| BUME_RS17195 | aquaporin family protein | 0.429 | -1.220 |
| BUME_RS00620 | Stp1/IreP family PP2C-type Ser/Thr phosphatase | 0.429 | -1.221 |
| BUME_RS14725 | 16S rRNA (adenine(1518)-N(6)/adenine(1519)-N (6))-dimethyltransferase RsmA | 0.429 | -1.222 |
| BUME_RS03520 | L-fuculose-phosphate aldolase | 0.429 | -1.222 |
| BUME_RS11265 | protein jag | 0.430 | -1.218 |
| BUME_RS13480 | helix-turn-helix domain-containing protein | 0.430 | -1.217 |
| BUME_RS06715 | DMT family transporter | 0.431 | -1.214 |
| BUME_RS15020 | ABC transporter substrate-binding protein | 0.431 | -1.214 |
| BUME_RS03615 | C39 family peptidase | 0.433 | -1.209 |
| BUME_RS16725 | glutamine-hydrolyzing GMP synthase | 0.433 | -1.207 |
| BUME_RS00770 | serine hydroxymethyltransferase | 0.433 | -1.207 |
| BUME_RS05775 | ATP-binding cassette domain-containing protein | 0.433 | -1.208 |
| BUME_RS18225 | response regulator transcription factor | 0.433 | -1.207 |
| BUME_RS06300 | patatin-like phospholipase family protein | 0.434 | -1.203 |
| BUME_RS04455 | hypothetical protein | 0.434 | -1.203 |
| BUME_RS09845 | amidohydrolase | 0.435 | -1.202 |
| BUME_RS14375 | DUF362 domain-containing protein | 0.435 | -1.202 |
| BUME_RS03790 | GntR family transcriptional regulator | 0.436 | -1.197 |
| BUME_RS05040 | TetR/AcrR family transcriptional regulator | 0.436 | -1.197 |
| BUME_RS15795 | 50S ribosomal protein L5 | 0.437 | -1.195 |
| BUME_RS16700 | zinc-binding dehydrogenase | 0.437 | -1.194 |
| BUME_RS06800 | hypothetical protein | 0.437 | -1.194 |
| BUME_RS02205 | ATP-binding protein | 0.437 | -1.194 |
| BUME_RS07320 | 50S ribosomal protein L10 | 0.438 | -1.193 |
| BUME_RS06030 | 1,4-alpha-glucan branching protein GlgB | 0.438 | -1.192 |
| BUME_RS09840 | molybdopterin molybdenumtransferase MoeA | 0.438 | -1.191 |
| BUME_RS19035 | helix-turn-helix domain-containing protein | 0.438 | -1.192 |
| BUME_RS07305 | transcription termination/antitermination protein NusG | 0.438 | -1.192 |
| BUME_RS10115 | DeoR/GlpR transcriptional regulator | 0.438 | -1.192 |
| BUME_RS09320 | MFS transporter | 0.438 | -1.190 |
| BUME_RS12065 | N-acetyltransferase | 0.438 | -1.190 |
| BUME_RS10690 | 3-deoxy-7-phosphoheptulonate synthase | 0.439 | -1.188 |
| BUME_RS15845 | 50S ribosomal protein L23 | 0.439 | -1.186 |
| BUME_RS08360 | sensor histidine kinase | 0.439 | -1.186 |
| BUME_RS09810 | response regulator transcription factor | 0.439 | -1.189 |
| BUME_RS00455 | Ig-like domain-containing protein | 0.440 | -1.184 |
| BUME_RS10125 | aldo/keto reductase | 0.440 | -1.183 |
| BUME_RS12270 | FadR family transcriptional regulator | 0.440 | -1.186 |
| BUME_RS00715 | hypothetical protein | 0.440 | -1.184 |
| BUME_RS12940 | phosphoribosylglycinamide formyltransferase | 0.440 | -1.185 |
| BUME_RS04025 | recombinase family protein | 0.440 | -1.185 |
| BUME_RS15925 | tyrosine--tRNA ligase | 0.441 | -1.181 |
| BUME_RS11285 | ParB/RepB/Spo0J family partition protein | 0.441 | -1.180 |
| BUME_RS20160 | preprotein translocase subunit SecE | 0.441 | -1.181 |
| BUME_RS12760 | 5'-nucleotidase | 0.441 | -1.183 |
| BUME_RS00760 | GlsB/YeaQ/YmgE family stress response membrane protein | 0.441 | -1.182 |
| BUME_RS13075 | hypothetical protein | 0.441 | -1.182 |
| BUME_RS04685 | leucine--tRNA ligase | 0.442 | -1.179 |
| BUME_RS15860 | 30S ribosomal protein S10 | 0.442 | -1.179 |
| BUME_RS00590 | primosomal protein N' | 0.442 | -1.176 |
| BUME_RS12295 | class I SAM-dependent methyltransferase | 0.442 | -1.179 |
| BUME_RS08875 | electron transfer flavoprotein alpha/beta-subunit | 0.442 | -1.179 |
| BUME_RS07975 | hypothetical protein | 0.442 | -1.177 |
| BUME_RS00690 | acetolactate synthase small subunit | 0.443 | -1.173 |
| BUME_RS18780 | selenide, water dikinase SelD | 0.443 | -1.175 |
| BUME_RS12605 | PTS sugar transporter subunit IIA | 0.443 | -1.173 |
| BUME_RS04480 | chitobiase/beta-hexosaminidase C-terminal domain-containing protein | 0.444 | -1.171 |
| BUME_RS17085 | UDP-glucose 4-epimerase GalE | 0.444 | -1.172 |
| BUME_RS14500 | HAD-IA family hydrolase | 0.444 | -1.173 |
| BUME_RS10220 | hypothetical protein | 0.444 | -1.173 |
| BUME_RS19100 | YifB family Mg chelatase-like AAA ATPase | 0.444 | -1.170 |
| BUME_RS01545 | TetR/AcrR family transcriptional regulator | 0.444 | -1.170 |
| BUME_RS09310 | hypothetical protein | 0.444 | -1.172 |
| BUME_RS05550 | phosphate ABC transporter permease subunit PstC | 0.444 | -1.171 |
| BUME_RS19385 | oligosaccharide flippase family protein | 0.444 | -1.173 |
| BUME_RS08710 | B12-binding domain-containing radical SAM protein | 0.445 | -1.168 |
| BUME_RS19355 | N-acetyltransferase | 0.445 | -1.169 |
| BUME_RS18830 | YgeY family selenium metabolism-linked hydrolase | 0.445 | -1.169 |
| BUME_RS15520 | hypothetical protein | 0.446 | -1.166 |
| BUME_RS15150 | histidine kinase | 0.446 | -1.166 |
| BUME_RS05205 | site-specific integrase | 0.446 | -1.164 |
| BUME_RS07520 | YhgE/Pip domain-containing protein | 0.446 | -1.166 |
| BUME_RS02225 | bifunctional enoyl-CoA hydratase/phosphate acetyltransferase | 0.447 | -1.161 |
| BUME_RS07845 | thioredoxin-disulfide reductase | 0.447 | -1.163 |
| BUME_RS18845 | putative aminohydrolase SsnA | 0.447 | -1.160 |
| BUME_RS06115 | GBS Bsp-like repeat-containing protein | 0.448 | -1.159 |
| BUME_RS06470 | MarR family transcriptional regulator | 0.448 | -1.158 |
| BUME_RS12440 | hypothetical protein | 0.448 | -1.159 |
| BUME_RS08900 | response regulator transcription factor | 0.448 | -1.159 |
| BUME_RS06535 | AraC family transcriptional regulator | 0.448 | -1.160 |
| BUME_RS10310 | choloylglycine hydrolase family protein | 0.449 | -1.155 |
| BUME_RS11725 | cell division protein FtsI | 0.449 | -1.154 |
| BUME_RS09075 | ABC-F type ribosomal protection protein | 0.449 | -1.156 |
| BUME_RS17235 | ABC transporter permease | 0.449 | -1.156 |
| BUME_RS15765 | 50S ribosomal protein L30 | 0.449 | -1.156 |
| BUME_RS05580 | LysR family transcriptional regulator | 0.449 | -1.156 |
| BUME_RS02670 | DUF1015 domain-containing protein | 0.450 | -1.152 |
| BUME_RS02690 | TIGR00159 family protein | 0.450 | -1.152 |
| BUME_RS00600 | methionyl-tRNA formyltransferase | 0.450 | -1.151 |
| BUME_RS04865 | GNAT family N-acetyltransferase | 0.450 | -1.150 |
| BUME_RS18755 | molybdate ABC transporter permease subunit | 0.450 | -1.153 |
| BUME_RS10685 | prephenate dehydrogenase | 0.451 | -1.150 |
| BUME_RS04610 | aminopeptidase P N-terminal domain-containing protein | 0.451 | -1.149 |
| BUME_RS10275 | hypothetical protein | 0.451 | -1.150 |
| BUME_RS15415 | ribosome small subunit-dependent GTPase A | 0.451 | -1.149 |
| BUME_RS07010 | tRNA guanosine(34) transglycosylase Tgt | 0.452 | -1.147 |
| BUME_RS17755 | hypothetical protein | 0.452 | -1.147 |
| BUME_RS06085 | acyltransferase | 0.452 | -1.144 |
| BUME_RS01240 | TlyA family RNA methyltransferase | 0.453 | -1.141 |
| BUME_RS20630 | hypothetical protein | 0.453 | -1.141 |
| BUME_RS13080 | LytTR family transcriptional regulator | 0.453 | -1.142 |
| BUME_RS20195 | GNAT family N-acetyltransferase | 0.453 | -1.141 |
| BUME_RS02460 | cobalt-precorrin 5A hydrolase | 0.453 | -1.142 |
| BUME_RS04655 | hypothetical protein | 0.454 | -1.138 |
| BUME_RS14485 | electron transfer flavoprotein subunit beta/FixA family protein | 0.454 | -1.141 |
| BUME_RS15995 | hypothetical protein | 0.454 | -1.139 |
| BUME_RS16210 | tRNA threonylcarbamoyladenosine dehydratase | 0.454 | -1.139 |
| BUME_RS02510 | class II fructose-1,6-bisphosphate aldolase | 0.455 | -1.137 |
| BUME_RS15625 | translational GTPase TypA | 0.455 | -1.135 |
| BUME_RS18890 | diguanylate cyclase | 0.455 | -1.135 |
| BUME_RS06325 | septation regulator SpoVG | 0.456 | -1.134 |
| BUME_RS15830 | 50S ribosomal protein L22 | 0.456 | -1.132 |
| BUME_RS06485 | NERD domain-containing protein | 0.456 | -1.132 |
| BUME_RS06455 | enoyl-CoA hydratase/isomerase family protein | 0.456 | -1.132 |
| BUME_RS18835 | diaminopropionate ammonia-lyase | 0.456 | -1.133 |
| BUME_RS14155 | DUF2703 domain-containing protein | 0.456 | -1.132 |
| BUME_RS17680 | V-type ATP synthase subunit K | 0.457 | -1.130 |
| BUME_RS13300 | hypothetical protein | 0.457 | -1.130 |
| BUME_RS02880 | hypothetical protein | 0.457 | -1.129 |
| BUME_RS14145 | helix-turn-helix transcriptional regulator | 0.458 | -1.126 |
| BUME_RS14450 | M20 family metallopeptidase | 0.458 | -1.126 |
| BUME_RS17275 | YhgE/Pip domain-containing protein | 0.459 | -1.122 |
| BUME_RS19120 | (d)CMP kinase | 0.459 | -1.125 |
| BUME_RS18750 | ATP-binding cassette domain-containing protein | 0.459 | -1.122 |
| BUME_RS10515 | HPr family phosphocarrier protein | 0.459 | -1.124 |
| BUME_RS00335 | TetR family transcriptional regulator | 0.459 | -1.123 |
| BUME_RS06140 | rhamnan synthesis F family protein | 0.460 | -1.122 |
| BUME_RS15840 | 50S ribosomal protein L2 | 0.460 | -1.120 |
| BUME_RS15855 | 50S ribosomal protein L3 | 0.460 | -1.120 |
| BUME_RS11175 | ABC transporter ATP-binding protein | 0.460 | -1.119 |
| BUME_RS00710 | FAD-dependent oxidoreductase | 0.461 | -1.117 |
| BUME_RS01280 | helix-turn-helix transcriptional regulator | 0.461 | -1.118 |
| BUME_RS13415 | radical SAM protein | 0.461 | -1.116 |
| BUME_RS19155 | segregation/condensation protein A | 0.462 | -1.115 |
| BUME_RS16165 | DUF3987 domain-containing protein | 0.462 | -1.115 |
| BUME_RS20220 | 50S ribosomal protein L34 | 0.462 | -1.115 |
| BUME_RS08195 | DUF616 domain-containing protein | 0.462 | -1.114 |
| BUME_RS20875 | hypothetical protein | 0.463 | -1.111 |
| BUME_RS14080 | hypothetical protein | 0.463 | -1.111 |
| BUME_RS11595 | MurR/RpiR family transcriptional regulator | 0.463 | -1.112 |
| BUME_RS01780 | hypothetical protein | 0.463 | -1.111 |
| BUME_RS19265 | 4Fe-4S dicluster domain-containing protein | 0.464 | -1.109 |
| BUME_RS18805 | transcriptional regulator | 0.464 | -1.108 |
| BUME_RS08355 | YhfC family intramembrane metalloprotease | 0.464 | -1.107 |
| BUME_RS18100 | HAMP domain-containing histidine kinase | 0.464 | -1.109 |
| BUME_RS20025 | trimethylamine methyltransferase family protein | 0.464 | -1.109 |
| BUME_RS13455 | 1-phosphofructokinase | 0.465 | -1.103 |
| BUME_RS12610 | hypothetical protein | 0.465 | -1.104 |
| BUME_RS17490 | Na+/H+ antiporter NhaC family protein | 0.466 | -1.101 |
| BUME_RS06205 | amino acid ABC transporter ATP-binding protein | 0.466 | -1.101 |
| BUME_RS01315 | helix-turn-helix transcriptional regulator | 0.466 | -1.101 |
| BUME_RS19990 | hypothetical protein | 0.466 | -1.102 |
| BUME_RS01035 | ribonuclease R | 0.467 | -1.097 |
| BUME_RS04550 | hypothetical protein | 0.467 | -1.100 |
| BUME_RS09755 | nitroreductase family protein | 0.467 | -1.097 |
| BUME_RS08885 | ABC transporter ATP-binding protein | 0.467 | -1.098 |
| BUME_RS13450 | PTS sugar transporter subunit IIA | 0.468 | -1.095 |
| BUME_RS15915 | class I SAM-dependent RNA methyltransferase | 0.468 | -1.096 |
| BUME_RS07645 | MBL fold metallo-hydrolase | 0.468 | -1.096 |
| BUME_RS12300 | YbaK/EbsC family protein | 0.468 | -1.096 |
| BUME_RS04945 | hypothetical protein | 0.469 | -1.094 |
| BUME_RS09305 | class I SAM-dependent methyltransferase | 0.469 | -1.094 |
| BUME_RS20125 | VanZ family protein | 0.469 | -1.092 |
| BUME_RS07290 | N-acetyltransferase | 0.469 | -1.093 |
| BUME_RS08775 | MerR family transcriptional regulator | 0.469 | -1.093 |
| BUME_RS05625 | deoxyribonuclease IV | 0.470 | -1.089 |
| BUME_RS03230 | response regulator | 0.470 | -1.090 |
| BUME_RS03775 | carboxylesterase family protein | 0.470 | -1.090 |
| BUME_RS11680 | class II fructose-bisphosphate aldolase | 0.470 | -1.089 |
| BUME_RS14175 | hypothetical protein | 0.470 | -1.090 |
| BUME_RS15675 | 30S ribosomal protein S9 | 0.471 | -1.086 |
| BUME_RS06805 | DUF2156 domain-containing protein | 0.471 | -1.086 |
| BUME_RS17620 | TetR/AcrR family transcriptional regulator | 0.471 | -1.087 |
| BUME_RS11960 | aspartate ammonia-lyase | 0.472 | -1.082 |
| BUME_RS05055 | cobalt ECF transporter T component CbiQ | 0.472 | -1.082 |
| BUME_RS10530 | PTS glucitol/sorbitol transporter subunit IIC | 0.473 | -1.079 |
| BUME_RS19500 | response regulator transcription factor | 0.473 | -1.081 |
| BUME_RS17700 | V-type ATP synthase subunit A | 0.474 | -1.078 |
| BUME_RS18870 | heavy metal translocating P-type ATPase | 0.474 | -1.077 |
| BUME_RS15850 | 50S ribosomal protein L4 | 0.474 | -1.077 |
| BUME_RS09560 | hypothetical protein | 0.474 | -1.076 |
| BUME_RS11215 | PTS sugar transporter subunit IIA | 0.474 | -1.078 |
| BUME_RS06020 | glucose-1-phosphate adenylyltransferase | 0.475 | -1.074 |
| BUME_RS18190 | ABC transporter substrate-binding protein | 0.475 | -1.074 |
| BUME_RS15070 | DMT family transporter | 0.475 | -1.074 |
| BUME_RS19790 | tRNA (adenosine(37)-N6)-dimethylallyltransferase MiaA | 0.476 | -1.072 |
| BUME_RS02445 | cobyric acid synthase | 0.476 | -1.071 |
| BUME_RS14505 | GHKL domain-containing protein | 0.476 | -1.071 |
| BUME_RS11965 | TetM/TetW/TetO/TetS family tetracycline resistance ribosomal protection protein | 0.476 | -1.071 |
| BUME_RS10840 | nucleotidyltransferase family protein | 0.476 | -1.071 |
| BUME_RS13595 | P-II family nitrogen regulator | 0.477 | -1.069 |
| BUME_RS15515 | DUF1211 domain-containing protein | 0.477 | -1.069 |
| BUME_RS09180 | ABC-2 transporter permease | 0.477 | -1.068 |
| BUME_RS03495 | hypothetical protein | 0.478 | -1.064 |
| BUME_RS11160 | FAD-dependent oxidoreductase | 0.478 | -1.065 |
| BUME_RS17050 | hypothetical protein | 0.478 | -1.064 |
| BUME_RS17670 | hypothetical protein | 0.479 | -1.062 |
| BUME_RS19260 | NAD(P)H-dependent oxidoreductase subunit E | 0.479 | -1.062 |
| BUME_RS10725 | helix-turn-helix transcriptional regulator | 0.479 | -1.063 |
| BUME_RS12235 | DUF1801 domain-containing protein | 0.479 | -1.061 |
| BUME_RS07500 | molecular chaperone DnaK | 0.480 | -1.059 |
| BUME_RS01585 | glutamate decarboxylase | 0.480 | -1.060 |
| BUME_RS17515 | MBL fold metallo-hydrolase | 0.480 | -1.058 |
| BUME_RS00635 | thiamine diphosphokinase | 0.480 | -1.057 |
| BUME_RS05615 | alpha-amylase | 0.480 | -1.060 |
| BUME_RS01955 | sigma 54-interacting transcriptional regulator | 0.480 | -1.058 |
| BUME_RS12225 | LPXTG cell wall anchor domain-containing protein | 0.480 | -1.059 |
| BUME_RS09760 | PadR family transcriptional regulator | 0.480 | -1.059 |
| BUME_RS18865 | RICIN domain-containing protein | 0.481 | -1.057 |
| BUME_RS12845 | ABC transporter permease | 0.481 | -1.057 |
| BUME_RS08070 | FAD-dependent oxidoreductase | 0.481 | -1.057 |
| BUME_RS04540 | CpsD/CapB family tyrosine-protein kinase | 0.482 | -1.053 |
| BUME_RS18520 | phenylacetate--CoA ligase | 0.482 | -1.054 |
| BUME_RS10900 | S8 family serine peptidase | 0.482 | -1.052 |
| BUME_RS07345 | zinc ribbon domain-containing protein | 0.482 | -1.052 |
| BUME_RS15865 | DegV family protein | 0.482 | -1.054 |
| BUME_RS13835 | formate dehydrogenase accessory sulfurtransferase FdhD | 0.483 | -1.051 |
| BUME_RS16320 | hypothetical protein | 0.483 | -1.050 |
| BUME_RS20575 | hypothetical protein | 0.483 | -1.049 |
| BUME_RS13460 | DeoR/GlpR transcriptional regulator | 0.483 | -1.049 |
| BUME_RS00420 | HD domain-containing protein | 0.483 | -1.051 |
| BUME_RS15930 | AI-2E family transporter | 0.484 | -1.048 |
| BUME_RS00930 | 4-hydroxy-tetrahydrodipicolinate reductase | 0.484 | -1.047 |
| BUME_RS12840 | ABC transporter permease | 0.484 | -1.046 |
| BUME_RS05530 | flavin reductase | 0.485 | -1.043 |
| BUME_RS04075 | type II toxin-antitoxin system HicB family antitoxin | 0.485 | -1.045 |
| BUME_RS01455 | sporulation integral membrane protein YtvI | 0.485 | -1.045 |
| BUME_RS19795 | DNA mismatch repair endonuclease MutL | 0.486 | -1.041 |
| BUME_RS03880 | nickel pincer cofactor biosynthesis protein LarB | 0.486 | -1.042 |
| BUME_RS00925 | 2,3,4,5-tetrahydropyridine-2,6-dicarboxylate N-acetyltransferase | 0.486 | -1.041 |
| BUME_RS11995 | HAMP domain-containing histidine kinase | 0.486 | -1.040 |
| BUME_RS00785 | insulinase family protein | 0.487 | -1.038 |
| BUME_RS05570 | fibronectin type III domain-containing protein | 0.487 | -1.037 |
| BUME_RS04645 | excinuclease ABC subunit UvrA | 0.488 | -1.035 |
| BUME_RS10675 | CRISPR-associated endoribonuclease Cas6 | 0.488 | -1.034 |
| BUME_RS00790 | serine hydrolase | 0.489 | -1.032 |
| BUME_RS17685 | V-type ATP synthase subunit E | 0.489 | -1.032 |
| BUME_RS07585 | ATP-dependent Clp endopeptidase proteolytic subunit ClpP | 0.489 | -1.032 |
| BUME_RS06170 | glycosyltransferase family 39 protein | 0.489 | -1.032 |
| BUME_RS19775 | TrkH family potassium uptake protein | 0.489 | -1.033 |
| BUME_RS01415 | FtsX-like permease family protein | 0.489 | -1.032 |
| BUME_RS13545 | GntR family transcriptional regulator | 0.489 | -1.031 |
| BUME_RS10185 | TIGR03915 family putative DNA repair protein | 0.489 | -1.033 |
| BUME_RS14445 | ammonium transporter | 0.489 | -1.033 |
| BUME_RS14150 | winged helix-turn-helix transcriptional regulator | 0.490 | -1.028 |
| BUME_RS16255 | ribose 5-phosphate isomerase B | 0.490 | -1.030 |
| BUME_RS15325 | energy-coupled thiamine transporter ThiT | 0.490 | -1.030 |
| BUME_RS02945 | type I glyceraldehyde-3-phosphate dehydrogenase | 0.491 | -1.027 |
| BUME_RS06080 | ABC transporter ATP-binding protein | 0.491 | -1.027 |
| BUME_RS08650 | TraB/GumN family protein | 0.491 | -1.026 |
| BUME_RS12340 | Fic family protein | 0.491 | -1.026 |
| BUME_RS04545 | capsular polysaccharide biosynthesis protein | 0.491 | -1.025 |
| BUME_RS03225 | GGDEF domain-containing protein | 0.491 | -1.026 |
| BUME_RS01630 | ATP-binding cassette domain-containing protein | 0.492 | -1.025 |
| BUME_RS13605 | DUF1538 domain-containing protein | 0.492 | -1.022 |
| BUME_RS07075 | FMN-binding domain-containing protein | 0.493 | -1.020 |
| BUME_RS12680 | Ltp family lipoprotein | 0.493 | -1.019 |
| BUME_RS12965 | phosphoribosylformylglycinamidine synthase | 0.493 | -1.021 |
| BUME_RS02200 | 4Fe-4S binding protein | 0.493 | -1.019 |
| BUME_RS09815 | two-component sensor histidine kinase | 0.493 | -1.021 |
| BUME_RS04100 | hypothetical protein | 0.493 | -1.020 |
| BUME_RS01195 | PAS domain-containing hybrid sensor histidine kinase/response regulator | 0.493 | -1.021 |
| BUME_RS02270 | glycerol kinase GlpK | 0.493 | -1.021 |
| BUME_RS02305 | SigB/SigF/SigG family RNA polymerase sigma factor | 0.494 | -1.018 |
| BUME_RS00740 | sensor domain-containing diguanylate cyclase | 0.494 | -1.018 |
| BUME_RS19660 | tRNA 4-thiouridine(8) synthase ThiI | 0.494 | -1.017 |
| BUME_RS07295 | 50S ribosomal protein L33 | 0.494 | -1.017 |
| BUME_RS12425 | hypothetical protein | 0.494 | -1.017 |
| BUME_RS09575 | GNAT family N-acetyltransferase | 0.494 | -1.016 |
| BUME_RS12100 | TetR/AcrR family transcriptional regulator | 0.495 | -1.016 |
| BUME_RS03010 | 1,4-alpha-glucan branching protein GlgB | 0.495 | -1.013 |
| BUME_RS11280 | 16S rRNA (guanine(527)-N(7))-methyltransferase RsmG | 0.495 | -1.014 |
| BUME_RS10935 | trimethylamine methyltransferase family protein | 0.495 | -1.014 |
| BUME_RS08865 | ATP-binding protein | 0.496 | -1.011 |
| BUME_RS11940 | lactate dehydrogenase | 0.496 | -1.012 |
| BUME_RS06955 | cobalamin synthesis protein P47K | 0.496 | -1.011 |
| BUME_RS11690 | triose-phosphate isomerase | 0.496 | -1.011 |
| BUME_RS17875 | GNAT family N-acetyltransferase | 0.496 | -1.012 |
| BUME_RS08830 | anthranilate synthase component I | 0.496 | -1.012 |
| BUME_RS18705 | V-type ATP synthase subunit B | 0.497 | -1.008 |
| BUME_RS00575 | YicC family protein | 0.497 | -1.009 |
| BUME_RS12140 | hypothetical protein | 0.497 | -1.008 |
| BUME_RS16730 | cysteine proteinase | 0.497 | -1.010 |
| BUME_RS14220 | arsenite efflux transporter metallochaperone ArsD | 0.497 | -1.009 |
| BUME_RS03695 | UDP-N-acetylmuramoyl-L-alanyl-D-glutamate--2,6-diaminopimelate ligase | 0.498 | -1.005 |
| BUME_RS04205 | aspartate kinase | 0.498 | -1.007 |
| BUME_RS13585 | EAL domain-containing protein | 0.498 | -1.007 |
| BUME_RS13380 | hypothetical protein | 0.498 | -1.007 |
| BUME_RS07570 | ABC transporter ATP-binding protein | 0.499 | -1.003 |
| BUME_RS09235 | phosphatidate cytidylyltransferase | 0.499 | -1.003 |
| BUME_RS11155 | Ig-like domain-containing protein | 0.499 | -1.002 |
| BUME_RS04880 | ribosome biogenesis GTPase YlqF | 0.499 | -1.004 |
| BUME_RS07005 | tRNA preQ1(34) S-adenosylmethionine ribosyltransferase-isomerase QueA | 0.499 | -1.002 |
| BUME_RS10190 | putative DNA modification/repair radical SAM protein | 0.499 | -1.002 |
| BUME_RS01150 | 2-dehydro-3-deoxyglucarate aldolase | 0.499 | -1.002 |
